# Architecture-Controllable Single-Crystal Helical Self-assembly of Small-Molecule Disulfides with Dynamic Chirality

**DOI:** 10.1021/jacs.3c00586

**Published:** 2023-03-06

**Authors:** Qi Zhang, Ryojun Toyoda, Lukas Pfeifer, Ben L. Feringa

**Affiliations:** † Stratingh Institute for Chemistry and Zernike Institute for Advanced Materials, University of Groningen, Nijenborgh 4, Groningen 9747 AG, The Netherlands; ‡ Department of Chemistry, Graduate School of Science, Tohoku University, 6-3 Aramaki-Aza-Aoba, Aobaku, Sendai 980-8578, Japan; § Laboratory of Photonics and Interfaces, Institute of Chemical Sciences and Engineering, School of Basic Sciences, Ecole Polytechnique Fédérale de Lausanne, Lausanne CH-1015, Switzerland; ∥ Key Laboratory for Advanced Materials and Joint International Research Laboratory of Precision Chemistry and Molecular Engineering, Feringa Nobel Prize Scientist Joint Research Center, Frontiers Science Center for Materiobiology and Dynamic Chemistry, Institute of Fine Chemicals, School of Chemistry and Molecular Engineering, East China University of Science and Technology, Shanghai 200237, China

## Abstract

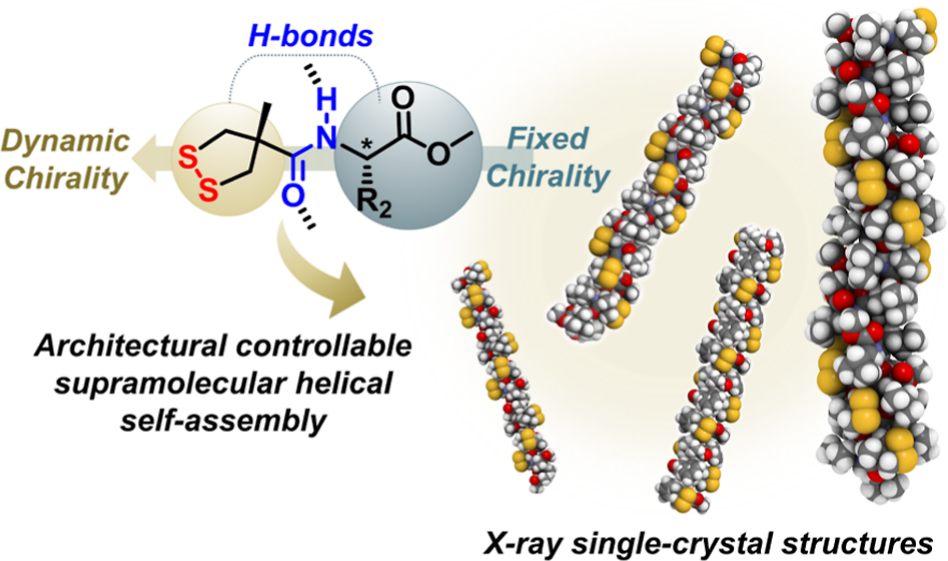

Beyond the common supramolecular helical polymers in solutions, controlling single-crystal helical self-assembly with precisely defined chirality and architectures has been challenging. Here, we report that simply merging static homochiral amino acids with dynamic chiral disulfides can produce a class of building blocks featuring supramolecular helical single-crystal self-assembly with unusual stereodivergency. Analysis of 20 single-crystal structures of 1,2-dithiolanes gives an atom-precision understanding of the chirality transfer from the molecular to supramolecular level, featuring homochiral and heterochiral helical supramolecular self-assembly in the solid state. The underlying structure–assembly relationship reveals that the synergistic interplay of intermolecular H-bonds and the 1,2-dithiolane ring with adaptive chirality plays a key role in determining the assembly pathway, also involving the effects of residue groups, substituents, molecular stacking, and solvents. The confinement effect in the solid state can stabilize the dynamic stereochemistry of disulfide bonds and selectively result in specific conformers that can minimize the energy of global supramolecular systems. We envision that these results represent a starting point to use dynamic chiral disulfide as a functional entity in supramolecular chemistry and may inspire a new class of supramolecular helical polymers with dynamic functions.

## Introduction

Molecular self-assembly, especially based on chiral molecules, is key to living systems featuring most intriguing architectures and functions.^1−3^ Proteins and DNA involve both H-bonded assemblies of homochiral building blocks, exhibiting the unique structural feature of supramolecular helicity (e.g., α-helix in proteins and double-helix in DNA).^4,5^ The underlying mechanism of how inherent (static) homochirality is controlled and transferred at (supra)molecular levels has been extensively explored in the past decades,^6−10^ including asymmetric organocatalysis,^6^ supramolecular polymers/gels,^7−9^ and liquid crystals.^10^ Despite established chirality transfer mechanisms in solvated environments, it still remains highly challenging to understand how chiral molecules, especially those featuring dynamic stereochemistry,^11−13^ assemble and deliver chiral information through solid-state supramolecular architectures.

Stereochemistry in solid-state materials is of great importance both from a fundamental and applied science perspective.^14−16^ The most famous example is Pasteur’s discovery in 1848 showing the separation of tartrate enantiomers by crystallization, which later opened the area of structural chemistry highly significant to organic chemistry and biochemistry.^17^ Prominent is also the role of chirality related to material science, i.e., the tactility effect of macromolecules.^18^ The relative stereochemistry of adjacent stereogenic units within a polymer remarkably determines the interchain interactions, crystalline degree, and thus material properties (e.g., melting point, glassy transition temperature, mechanical performance, thermal stability).^19^ The fundamental stereochemical principles based on static chiral molecules have been reasonably well understood. On the other hand, while translating and expressing static chirality, many natural (macro)molecular entities also feature complicated dynamic chirality (e.g., left-handed and right-handed DNA duplexes),^20^ resulting in multifaceted systems with stereodiversity originated from homochirality.^1^ However, unveiling the underlying chirality transfer mechanism of self-assembled architectures from dynamic chiral entities remains challenging, especially based on single-crystal structures with atom precision.

**Figure 1:**
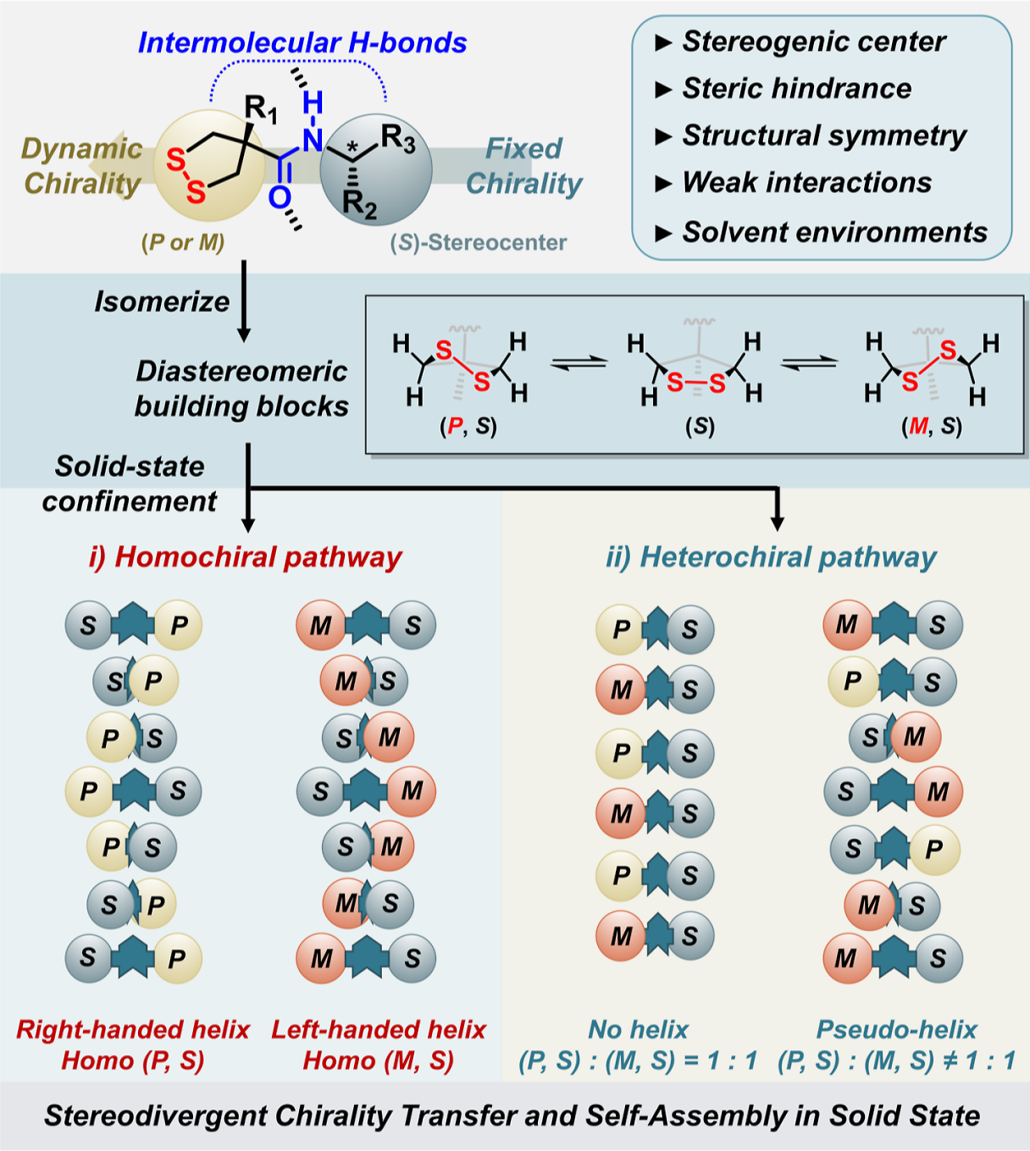
Conceptual illustration of the multi-pathway self-assembly of chiral 1,2-dithiolanes.

Here, we report systematic structural insights into the single-crystal self-assembly of a series of 1,2-dithiolanes, exhibiting a subtle interplay between dynamic chiral disulfide bonds and amino acids with fixed chirality. Since the flipping motion of 1,2-dithiolane rings exhibits a low energy barrier in solution,^21^ the stereochemistry of the disulfide bond is dynamic and quickly racemizing at room temperature. Upon crystallization into the solid state, the resulting close molecular packing confines the flipping space and thus produces static helical chirality (*P*/*M*) in solid states (Figure 1). The analysis and comparison of the X-ray single-crystal structure of 20 disulfide derivatives showed highly structure-dependent H-bonding assemblies, which are strongly related to the dynamic chiral conformation of the disulfide five-membered rings in the solid states (Figure 1). The single crystal analysis of the supramolecular architectures reveals two stereodivergent pathways, featuring homochiral and heterochiral assemblies of the chiral supramolecular architectures. It should be noted that these stereochemical features (e.g., heterochiral assembly) are only applicable in the solid state instead of solution. As a result, a series of unprecedented supramolecular helical architectures in single crystals are constructed with controlled helicity and geometries due to the subtle interplay of several parameters in the intermolecularly H-bonded supramolecular systems (Figure 1). We foresee that this study will introduce a disulfide bond as a dynamic chiral entity and control element in the toolbox of supramolecular chemistry and materials.

## Results and Discussion

### Molecular Design and Principles

This research was based on our long-term exploration of disulfide-based dynamic chemistry and materials.^22−24^ We serendipitously found two unusual single-crystal architectures formed by simple chiral amides derived from methyl asparagusic acid (MAA) and left-handed amino acid methyl esters.^25^ One (MAA-l-Ala) shows a P-type supramolecular helix, while the other (MAA-l-*t*-Leu) forms an M-type helix (Table 1). This is intriguing from a stereochemical perspective because (i) helical single-crystal self-assembly is rare,^26−29^ especially based on such a simple molecule; and (ii) not only disulfide stereochemistry but also the supramolecular helicity of the assemblies can be tuned by residue groups, whereas the chiral source is the same left-handed amino acids. However, the underlying chirality transfer principle in the present system remains unclear: how does the static chirality of amino acids govern the dynamic chiral elements (i.e., disulfide bonds and supramolecular helicity) with a strong dependency on residue groups (the intrinsic molecular information)? Answering this fundamental question might introduce 1,2-dithiolane as a new dynamic chiral unit to design architecture-controlled helical supramolecular self-assembly with atomic precision.

**Table 1:**
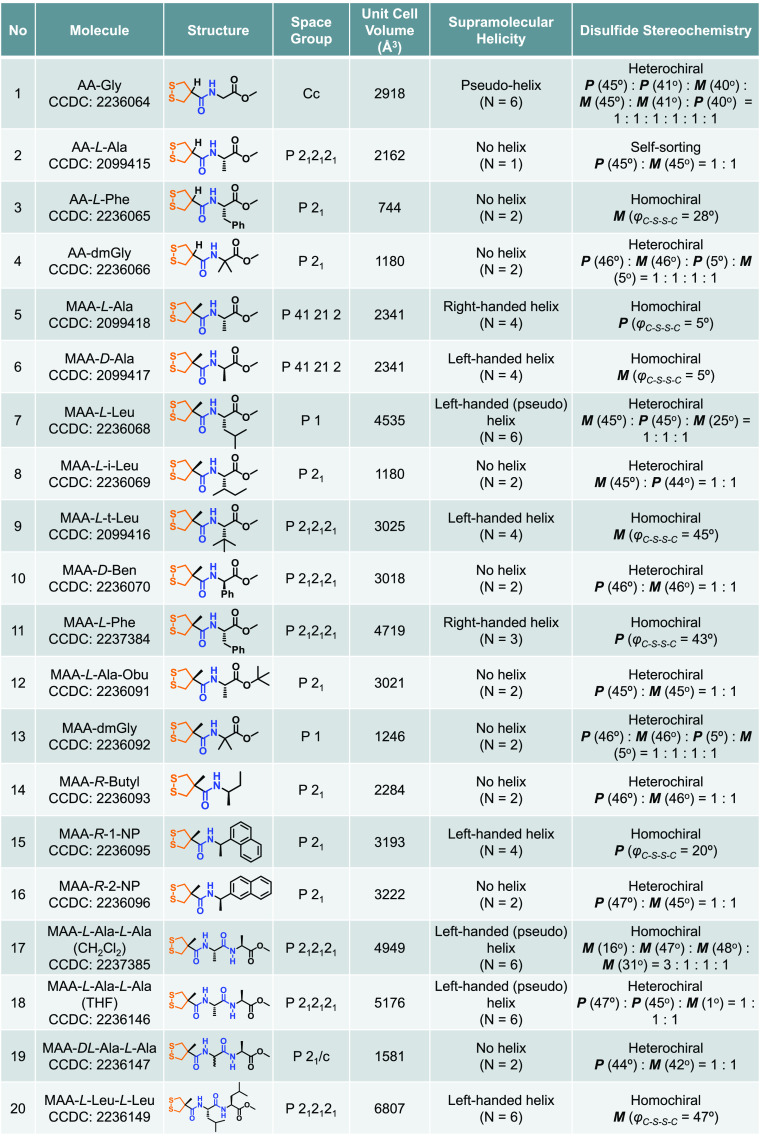
Summary for the X-Ray Single-Crystal Data of the Building Blocks^a^

a Supramolecular helicity and disulfide stereochemistry are shown for comparison. *N* number refers to the smallest repeating molecular units along the H-bond direction. In a few cases of disorder, the major geometry is used for assigning disulfide stereochemistry.

The substituent effect is known as one of the key factors that determine the molecular geometry, chirality transfer, and self-assembly in supramolecular systems, from which the complexity of protein architectures originates.^3^ Here, we aim to systematically investigate the substituent effect at two positions of this simple molecular skeleton, including the methyl substituent (R_1_) on the 1,2-dithiolane ring and the diverse groups (R_2_, R_3_) at the carbon center (Figure 1). Toward this goal, a series of compounds (Table 1) were synthesized by typical amide coupling reactions using commercially available enantiomerically pure chiral amines as starting materials and 1-ethyl-3-(3-dimethylaminopropyl) carbodiimide (EDC)/hydroxybenzotriazole (HOBt) as the coupling reagent (see experimental details and molecular characterizations in Supporting Information). All the compounds were crystallized by direct solvent evaporation methods under dark and low-temperature conditions to avoid unwanted polymerization. The crystal structures have been summarized in Table 1 and individually presented in the Supporting Information (Figures S1–S19).

### Substituent Effect and Homochiral Self-Assembly

**Figure 2:**
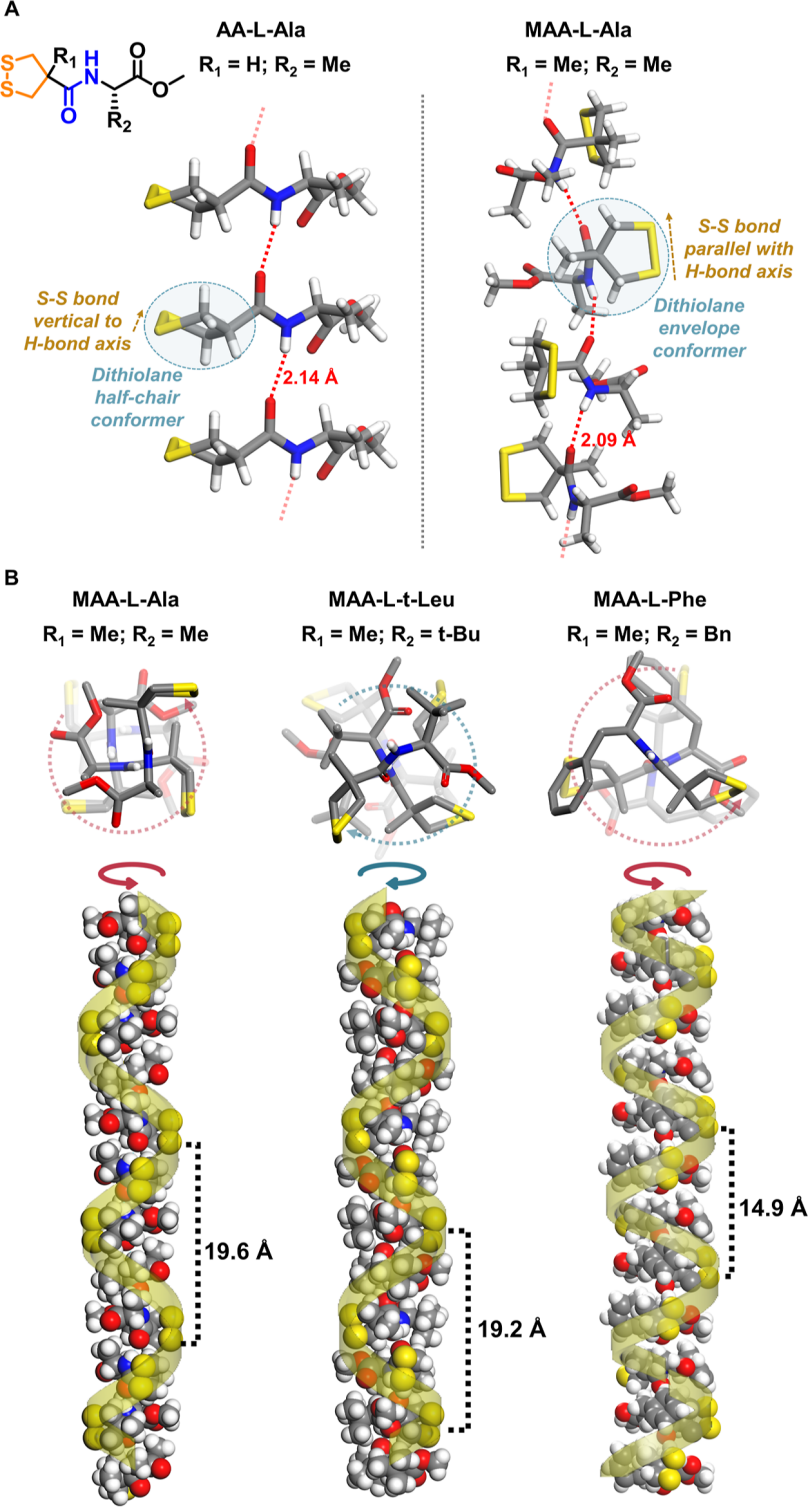
Architecture control of homochiral helical self-assembly. (A) Comparison of AA-l-Ala (left) and MAA-l-Ala (right) showing the methyl substituent effect of R_1_; (B) three examples of homochiral helical self-assembly showing the substituent effect of R_2_.

First, the substituent effect of the methyl group on the 1,2-dithiolane ring was investigated. Four molecules based on asparagusic acid (AA) (i.e., AA-Gly, AA-l-Ala, AA-l-Phe, and AA-dm-Gly) showed remarkably different assembled structures compared with their methyl-substituted analogues (i.e., MAA-l-Ala, MAA-l-Phe, and MAA-dm-Gly). For example, MAA-l-Ala exhibited homochiral *P*-helix self-assembly, while no helix was observed in the case of AA-l-Ala. The crystal structure of MAA-l-Phe showed a homochiral *P*-helix, while that of AA-l-Phe followed the heterochiral assembly and lacked supramolecular helicity in the crystal structure. By comparing the molecular geometry, especially the relative orientation of 1,2-dithiolane rings in MAA-based (R_1_ = Me) and AA-based (R_1_ = H) analogues (Figure 2A), we found that the presence of the R_1_ methyl group consistently led to the plane of the five-membered rings more parallel with the amide–amide H-bonding axis, while the cases of AA-based molecules were opposite (i.e., vertical to the H-bond axis). This geometry difference became more evident when realizing that the methyl substitute is at the α-position of the amide carbonyl groups and that the neighboring methyl substituent can significantly affect H-bonding interactions.^8^ Thus, the five-membered ring tends to adapt its orientation toward the parallel axle direction to minimize the steric hindrance caused by the “overcrowded” H-bond stacking to achieve global energy minimization.

Having related the methyl substituent effect (R_1_ = Me) with the ring orientation, it seems that this is not the only factor determining the homochiral/heterochiral self-assembly because not all the MAA-based chiral amides showed homochiral helical self-assembly. The substituent effect on the chiral amine side is also a key parameter. Focusing on the amino-acid-based MAA-analogues, we compared eight molecules with different substitute sizes and varying steric hindrances, including MAA-l-Ala, MAA-l-Leu, MAA-l-*i*-Leu, MAA-l-*t*-Leu, MAA-*D*-Ben, MAA-l-Phe, MAA-l-Ala-OBu, and MAA-dmGly (Table 1; Figures S1,S7–S13). Among them, MAA-l-Ala, MAA-l-*t*-Leu, and MAA-l-Phe exhibited homochiral helical self-assembly (Figure 2B), while others resulted in heterochiral assemblies, i.e., mixtures of (*P*, *S*) and (*M*, *S*) conformers in crystal structure (Table 1). Interestingly, the homochiral helical architectures of MAA-l-Ala, MAA-l-*t*-Leu and MAA-l-Phe showed geometrical differences (Figure 2B). The helical strand of MAA-l-Ala features a helical pitch of 19.6 Å containing four molecular units with homochiral *P*-type supramolecular helicity (homo-*P*(4)). The supramolecular architecture of the homochiral assemblies can be tuned by the R_2_ residue group: the crystal structure of MAA-l-*t*-Leu showed homochiral M-type supramolecular helicity with four molecular units as a repeating pitch (homo-*M*(4)), while MAA-l-Phe assembled into a homochiral P-type supramolecular helix featuring a shorter pitch (14.9 Å) with three molecular units (homo-*P*(3)) (Figures 2B and S11). These results indicate the architectural controllability that can be achieved in the single-crystal self-assembly of these 1,2-dithiolane-based chiral building blocks.

Further structure–assembly relationship is reflected by comparisons among a series of analogues in Table 1. Even the terminal ester group, which seems irrelevant to the H-bonding self-assembly, also affects the self-assembly process: replacing the methyl ester of MAA-l-Ala with *t*-butyl ester (i.e., MAA-l-Ala-OBu) led to heterochiral self-assembly instead of homochiral supramolecular helical self-assembly in the solid state (Figure S12). In view of the high symmetry in the homochiral cases (Figure 2), it is reasonable to infer that one key structural feature enabling homochiral self-assembly is the capability of 1,2-dithiolanes to produce a sterically “symmetric” environment along the H-bonding axis. Here, the mentioned “capability” means the potential of the five-membered ring to adapt its conformation to achieve the global energy minimum of the whole supramolecular system, even in some cases where planar disulfide bonds are found (e.g., φ_C-S-S-C_ = 5° in the crystal structure of MAA-l-Ala), which is enthalpically unfavored but is counteracted by intermolecular H-bonds and supramolecular stacking.

**Figure 3:**
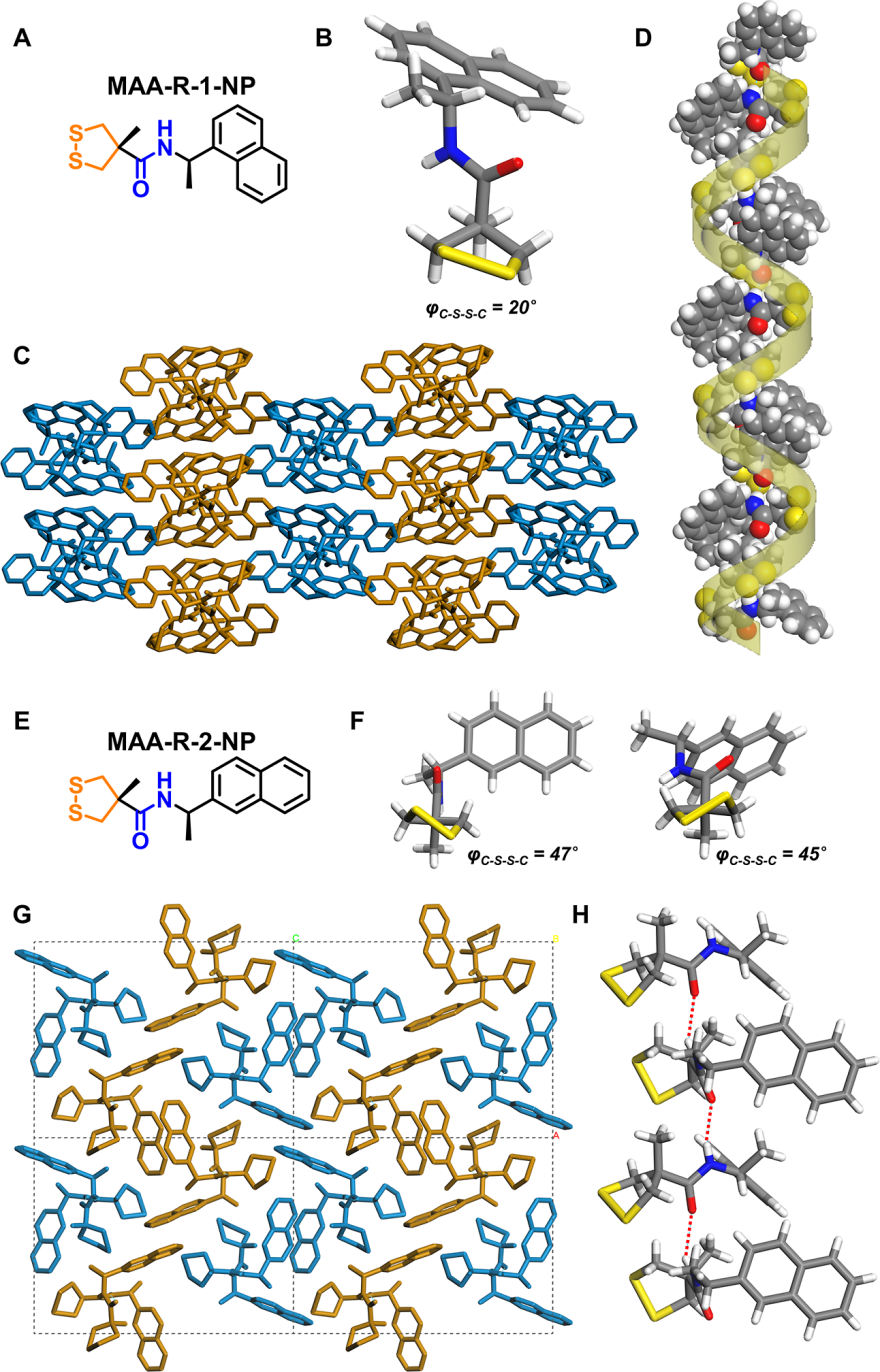
Substituent-dependent self-assembly of two amide analogues. (A-–D) Molecular structure (A) and crystal structure (B–D) of MAA-*R*-1-NP; (E–H) molecular structure (E) and crystal structure (F–H) of MAA-*R*-2-NP. In crystal packing patterns, the H-bonded arrays are marked with cyan and brown colors to readily distinguish the anti-parallel packing manner.

The homochiral helical self-assembly is not only limited for amino-acid derivatives but also observed for other chiral amines, suggesting the generality of this self-assembly system. Single-crystal structures of three chiral amides were obtained (MAA-*R*-butyl, MAA-*R*-1-NP, and MAA-*R*-2-NP). Among them, MAA-*R*-1-NP showed homochiral self-assembly, while others followed heterochiral assembly. Illustrative of this interesting phenomenon is the comparison of two isomers, MAA-*R*-1-NP and MAA-*R*-2-NP: the different substituent position in the naphthalene ring results in two self-assembly pathways and chirality transfer. MAA-*R*-1-NP presents anti-parallel distinct homo-*P*(4) helixes in the crystal structure (Figure 3), and the disulfide stereochemistry shows homo-*P* chirality (φ_C-S-S-C_ = 20°). In contrast, the crystal structure of MAA-*R*-2-NP showed no helix and heterochiral packing, with the equal presence of *P*-/*M*-chirality disulfide bonds. Notably, an edge-to-plane aromatic π–π stacking was observed between the neighboring two MAA-*R*-1-NP molecules along the H-bonding strands, while no favorable geometry of aromatic interactions was visible among the molecules at different H-bonding strands, indicating that the secondary side-by-side packing of the helical strands mainly relied on the space-filling weak interactions (e.g., van der Waals forces).

### Heterochiral Self-assembly

In general, it is recognized that over 90% of crystals are “heterochiral” (i.e., racemates) because a pair of enantiomers can form closer intermolecular packing in solid states due to spatial symmetry.^30−33^ In our study, the examples we denote as “heterochiral self-assembly” are unconventional because they are solid-state-confined diastereoisomeric conformers from molecules that are enantiopure in solutions because of the dynamic chiral 1,2-dithiolane units.

**Figure 4:**
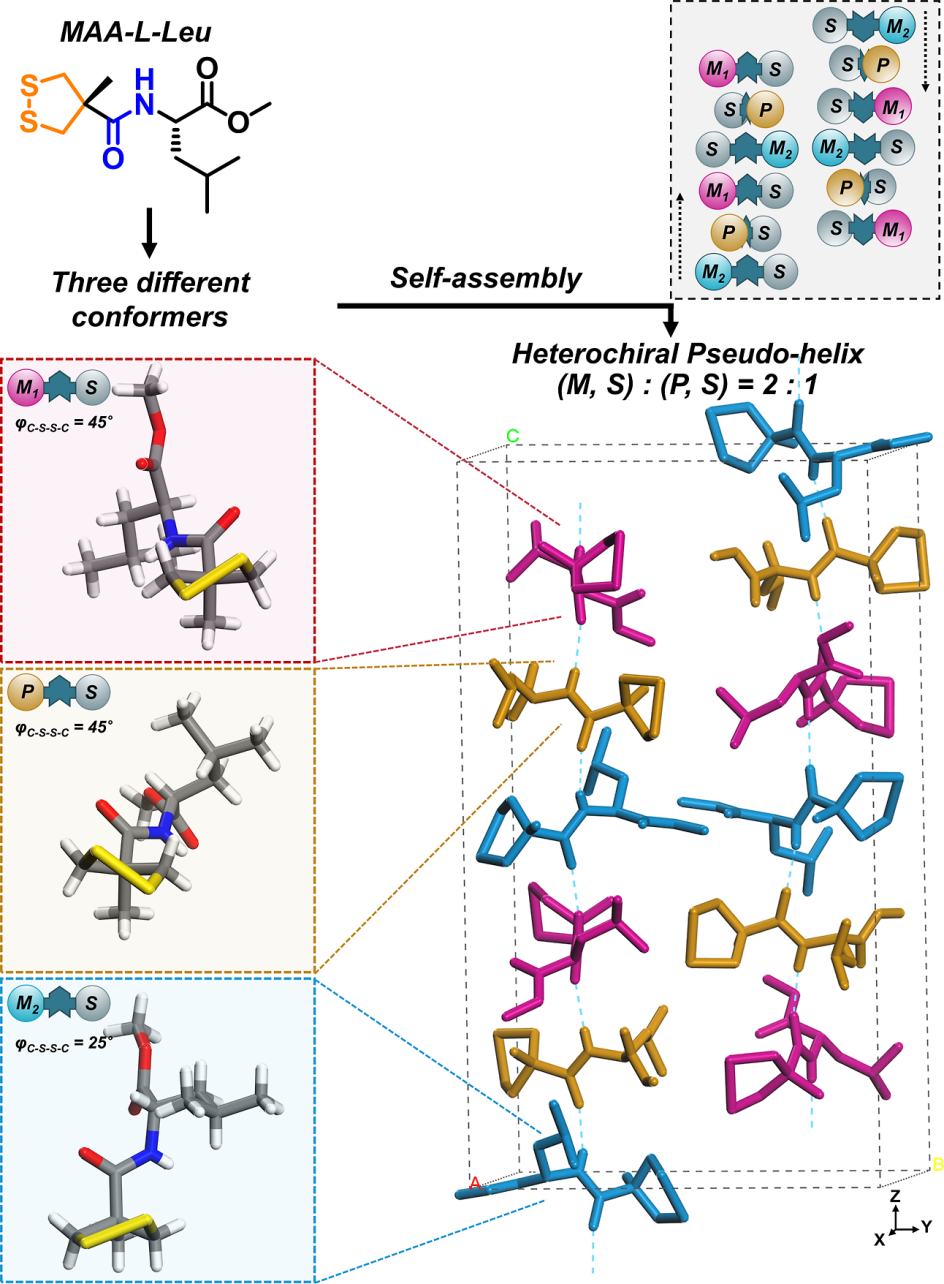
Heterochiral self-assembly of MAA-l-Leu in crystal architectures. In this case, three conformers (respectively marked by pink, orange, and cyan) co-assembled into pseudo-helical strands consisting of six molecules as a repeating unit. The strands also followed an antiparallel side-by-side secondary packing arrangement to result in structural symmetry in the solid state.

Taking MAA-l-Leu as an example, its single-crystal structure showed an unprecedented heterochiral assembly. The enantiopure MAA-l-Leu molecules assembled into pseudo-helical anti-paralleled H-bonding strands (Figures 4 and S7). Three different conformers were observed: two have (*M*, *S*) and one has (*P*, *S*) chirality. The two (*M*, *S*) conformers bear different disulfide dihedral angles (45 and 25°, respectively). Along the amide–amide H-bonding axis, *M*-helix-like molecular twisting was seen, despite the non-identical twisting angle among the neighboring units. Therefore, in this very special case of MAA-l-Leu, an unprecedented chirality transfer phenomenon was observed: the single-handedness chirality at the stereogenic center was delivered and expressed by the dynamic chirality of disulfide bonds into diastereoisomeric conformers with a ratio of *M*/*P* = 2:1. This is unusual because known small-molecule crystals constitute to be either racemates or conglomerates due to the requested high symmetry in crystal environments.^30^ At least three factors should be responsible for this unusual heterochiral system, e.g., (i) dynamic stereochemical adaptation of 1,2-dithiolanes to give the most favorable conformers for intermolecular packing, (ii) a subtle interplay related to steric hindrance occurring at both sides of the H-bonding axis, (iii) amide-mediated H-bonding interactions with anti-parallel direction to enhance symmetry at supramolecular level.

**Figure 5:**
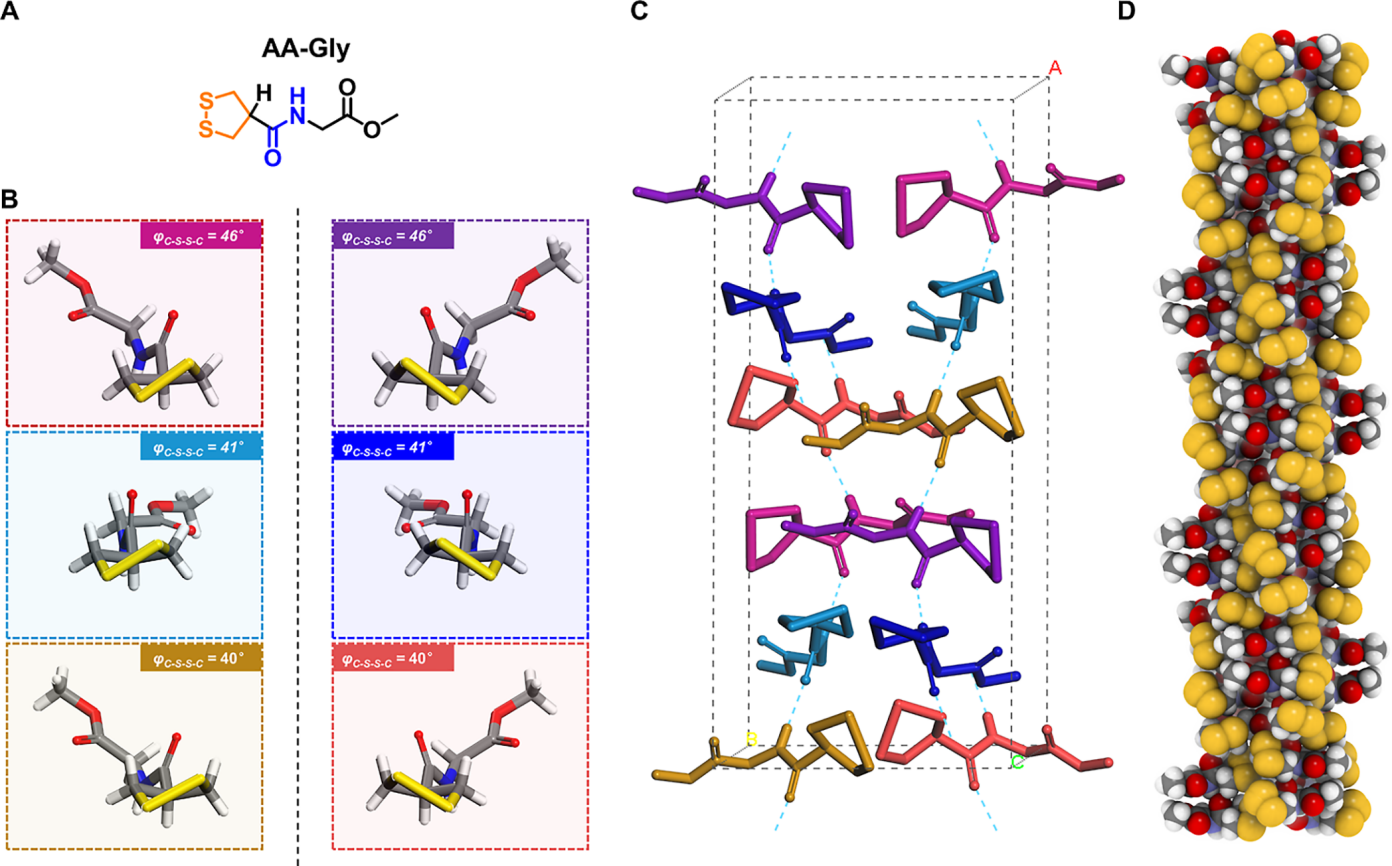
X-ray crystal structure of an achiral molecule AA-Gly exhibiting pseudo-helical patterns. (A) Molecular structure of AA-Gly; (B) six different conformers extracted from single-crystal structures; (C) unit cell of the crystal; (D) CPK model of the pseudo-helical pattern along the H-bond direction. The conformers in the solid states are *P*/*M* = 50/50.

Another surprising example of heterochiral self-assembly was observed in the single-crystal structure of AA-Gly (Figure 5). The “simplest” entity of the molecules investigated in this study, bearing no static stereogenic center, assembled into a pseudo-helical pattern with a polar space group (*Cc*) and high complexity (12 asymmetric units in a single unit cell with a unit volume of 2918 Å^3^). This achiral molecule folds into three sets of conformers, which co-assemble into H-bonding strands and are then stacked in an anti-paralleled manner, thus resulting in a complex pattern (Figure 5D). Comparing AA-Gly with AA-l-Ala, it is revealed that subtracting the static stereocenter going from alanine to glycine, in contrast, leads to higher complexity in the resulting supramolecular self-assembly, which reveals a subtle interplay between the molecular symmetry and assembling geometry in the solid state. The observations in this example are reminiscent of both the classic crystallization of achiral sodium perchlorate and the phenomenon of attrition-enhanced deracemization,^34,35^ which might inspire future explorations of generating homochirality by using this simple and achiral small molecule.

### Dipeptide Self-assembly

Having investigated the homochiral and heterochiral self-assembly of amides based on single amines, we also crystallized diamide analogues because the introduction of dipeptide could remarkably increase the complexity of static chiral units.^36−38^ Notably, here we do not aim at presenting a comprehensive screening regarding the structure–assembly relationships. We envision that the concept in the proposed supramolecular models in this study, including homochiral and heterochiral self-assembly, also applies to the double H-bonding self-assembly and aims to further expand the chemical spaces from single amides to dipeptide analogues.

The “simplest” dipeptide with two stereogenic centres (i.e., l-Ala-l-Ala) was coupled with MAA, giving enantiopure MAA-l-Ala-l-Ala. Unlike all the above crystals with no solvent co-crystallized, it appears that MAA-l-Ala-l-Ala easily co-assembles with solvents to crystallize. We managed to obtain two different crystals of MAA-l-Ala-l-Ala by solvent evaporation from the mixture solutions of tetrahydrofuran (THF)/heptane and CH_2_Cl_2_/heptane, respectively. Both of crystal architectures contained co-assembled solvent molecules (THF or CH_2_Cl_2_) (Figures S15 and S16). The single-crystal architectures showed that the dipeptide moieties assembled in a β-sheet-like manner, featuring a highly twisted geometry. Both of the two helical strands bear six molecules as a repeating unit and followed the anti-paralleled secondary stacking arrangement. Interestingly, the geometry of disulfide bonds in the two crystals showed divergency, meaning that the “minor” space-filling effect of external solvents, without intermolecular H-bond, made a remarkable difference in the global energy minimization of supramolecular self-assembly in crystal environments. This observation suggests that solvent environments, even non-binding cases, can also subtly affect the stereochemical information of disulfide bonds in noncovalent environments. The reflected principle is reminiscent of the earlier example of proline crystallization in chloroform and methanol,^39^ which leads to a key mechanism for chiral amplification.

**Figure 6:**
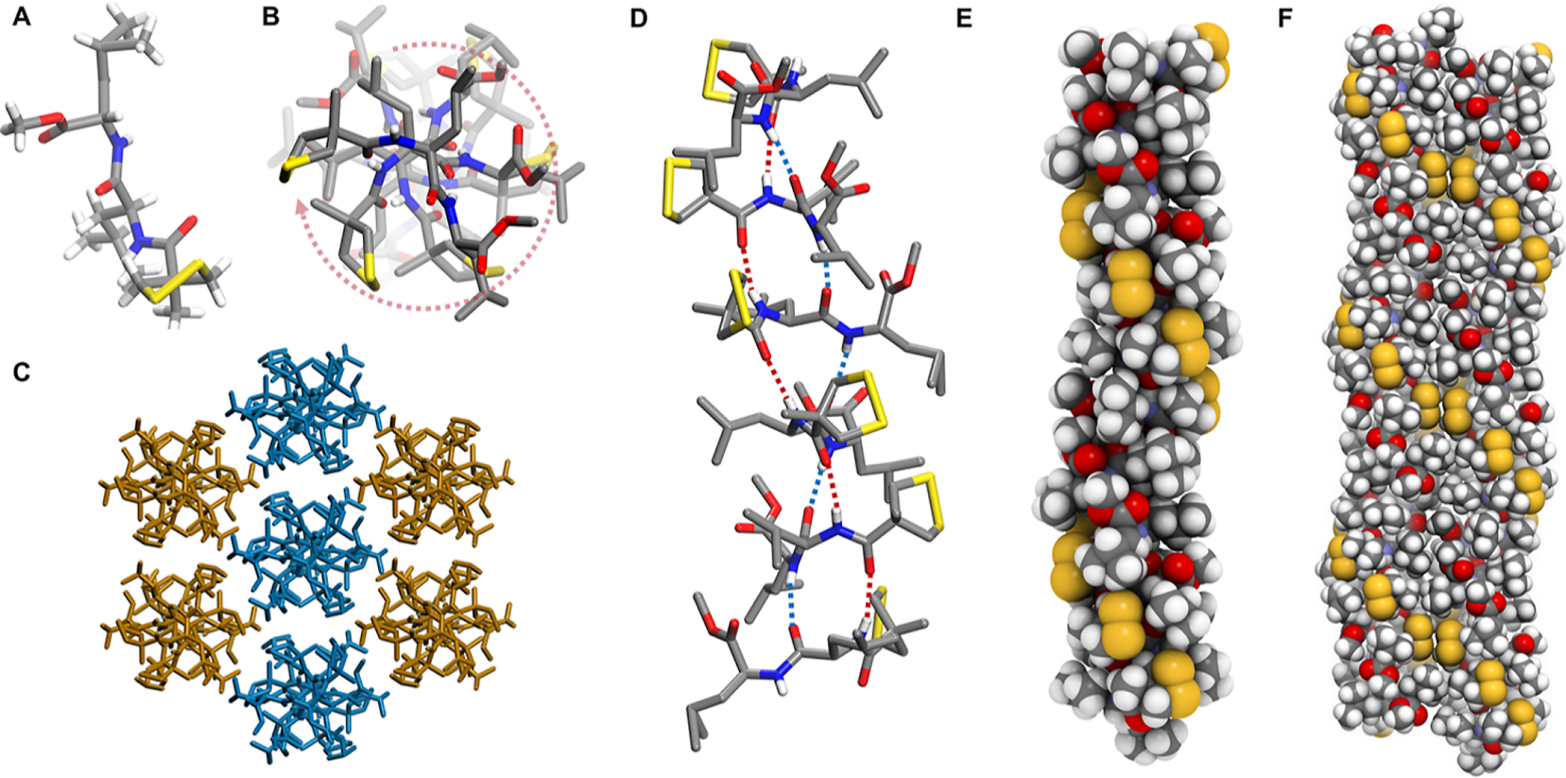
Homochiral helical single-crystal architectures of MAA-l-Leu-l-Leu. (A) Homochiral molecular structure in the solid state; (B) top view of a helical strand showing the anti-clockwise rotation with 60° between every two neighboring molecules; (C) anti-parallel crystal packing of the homochiral helical strands. Blue and orange colors are used to distinguish the two different directions of the strands; (D) twisted β-sheet-like H-bonding interactions in a six-mer assembly as the smallest repeating unit in the crystal architecture; (E) CPK model of a single helical strand; (F) side-by-side secondary packing manner of the two neighboring anti-paralleled helical strands.

Finally, we managed to obtain a single-crystal structure of MAA-l-Leu-l-Leu, which exhibited a homochiral helical self-assembly to form a homo-*M*(6) helix (Figure 6). No solvent was observed in the crystal structure. The molecules exhibited identical conformations with *M*-chiral disulfide bonds with a dihedral angle of 47°. At the supramolecular level, every six molecules constituted a repeating pitch of the helix along the H-bond growth direction, forming a very unusual β-sheet-like H-bond pattern with twisted geometry. The primary homochiral supramolecular helixes further self-assembled into the three-dimensional architecture by following an antiparallel side-by-side stacking manner. Compared to the homochiral helixes formed by single H-bonds (Figures 2 and 3A), this supramolecular helix formed by MAA-l-Leu-l-Leu bears a large helical pitch (27.2 nm). Further circular dichroism spectroscopic studies showed that the concentrated solution of MAA-l-Leu-l-Leu exhibited a strong negative band at 314 nm (Figures S18 and S19), attributed to the *M*-chiral disulfide bonds consistent with the stereochemistry in solid states. Considering the broad interest of dipeptide-based supramolecular materials,^36−38^ this dipeptide-based single-crystal self-assembly with homochiral supramolecular helicity might provide a distinctive model for further elaboration of functional chiral nanomaterials in the future.^14^

## Conclusions

In summary, we present a series of single-crystal H-bonding self-assembly structures based on 1,2-dithiolanes. A subtle interplay was discovered controlled by static stereogenic centers and dynamic chiral 1,2-dithiolane units, in which molecular geometry and symmetry play a key role in determining the H-bonding self-assembly. A key structural feature of the helically assembled molecules is that the orientation of the disulfide bond is (pseudo-)paralleled to the H-bonding direction, which provides a favorable packing geometry to facilitate the formation of helical organization along the H-bond axis. The supramolecular helicity shows a direct correlation with the disulfide stereochemistry in the solid state. All the helically assembled architectures bear homochiral disulfide bonds, whose stereochemistry (e.g., chirality and dihedral angle) shows a high dependency on the residue groups of amino acids as a result of several structural factors (e.g., molecular symmetry, steric hindrance, H-bonding strength, etc.). Introducing dipeptide enables the generation of helically twisted β-sheet-like supramolecular assemblies, which bear a helical pitch with every six molecules as the repeating unit. This discovery will open up a new avenue by introducing 1,2-dithiolane as a dynamic chiral control element in the study of amino acids, dipeptides, supramolecular self-assembly, and chiral materials. The underlying principles of chirality transfer and supramolecular self-assembly in the solid state might provide potential insights for research into the origin of life especially with regard to sulfur-containing biomolecules in prebiotic chemistry.^40,41^

## Supporting Information Available

Experimental details on the synthesis and structural characterizations of compounds, NMR spectra, CD spectra, and crystallographic data (PDF)
