# Supplementary material for: Architecture-Controllable Single-Crystal Helical Self-assembly of Small-Molecule Disulfides with Dynamic Chirality
Source: J Am Chem Soc. 2023 Mar 6;145(12):6976–85. doi: 10.1021/jacs.3c00586 (PMC10064337; doi:10.1021/jacs.3c00586)
Supplement: Supplementary file 1 — ja3c00586_si_001.pdf [file ja3c00586_si_001.pdf]

Supplementary Information for

Architecture-controllable single-crystal helical self-assembly of small-molecule disulfides with dynamic chirality

Qi Zhang[1]*, Ryojun Toyoda[1,2], Lukas Pfeifer[1,3], Ben L. Feringa[1,4]*

[1] *Stratingh Institute for Chemistry and Zernike Institute for Advanced Materials, University of Groningen, Nijenborgh 4, 9747 AG, Groningen, The Netherlands.*

[2] *Department of Chemistry, Graduate School of Science, Tohoku University 6-3 Aramaki-Aza-Aoba, Aobaku, Sendai 980-8578, Japan.*

[3] *Laboratory of Photonics and Interfaces, Institute of Chemical Sciences and Engineering, School of Basic Sciences, Ecole Polytechnique Fédérale de Lausanne, CH-1015 Lausanne, Switzerland.*

[4] *Key Laboratory for Advanced Materials and Joint International Research Laboratory of Precision Chemistry and Molecular Engineering, Feringa Nobel Prize Scientist Joint Research Center, Frontiers Science Center for Materiobiology and Dynamic Chemistry, Institute of Fine Chemicals, School of Chemistry and Molecular Engineering, East China University of Science and Technology, Shanghai 200237, China.*

*Corresponding author. Email: qi.zhang@rug.nl; b.l.feringa@rug.nl

Table of Contents:

Materials and Methods

Figs. S1 to S52

References (*S1*-*S6*)

Materials and Methods

Materials

All the reagents were obtained from commercial sources (Sigma-Aldrich, TCI, FluoroChem) and used as received without further purification. Solvents used for reactions were HPLC grade. Solvents used for spectroscopic experiments were spectroscopic grade (TCI) or NMR grade (Sigma-Aldrich). Solvents for NMR spectroscopy were used as received from Sigma-Aldrich. TLC plates were used to trace the reactions and visualized by iodine silica bath. Column chromatography was performed using Silica 90 Å as the stationary phase.

Instruments and Methods

NMR spectra

All the NMR spectra were recorded on a Varian Unity Plus 500 spectrometer (500 MHz or 400 MHz). Chemical shifts were denoted in *δ* values (*ppm*) relative to $CDCl_3$ (^1H: δ = 7.26; ^{13}C: δ = 77.00).

ATR IR spectra

All the ATR IR spectra were collected on a Perkin-Elmer FT-IR Spectrometer 400. A few drops of sample solution was added onto the surface of sample platform to evaporate the solvents at ambient conditions. The background of the sample platform was corrected. Unless stated otherwise, the temperature was room temperature (25°C).

Single crystal preparation

The single crystals were prepared by slow evaporation of the solvent at low temperature (0 ~ 4°C). The sample powders were dissolved in good solvents (e.g. CH_2Cl_2, diethyl ether) to obtain homogeneous yellow solution. Then 1 vol. equivalent amount of heptane was added and mixed. The resulting homogeneous solution was filtrated two times by cotton filter and then transferred into glass vials, which were sealed by para film with a few small holes. Then the vials were placed into a ventilated fridge to evaporate the solvents slowly. The crystals can be collected after 2 ~ 3 days and then stored in dark for measurement.

X-ray single crystal analysis

A single crystal sample was mounted on top of a cryoloop and transferred into the cold nitrogen stream (100 K) of a Bruker-AXS D8 Venture diffractometer. Data collection and reduction was performed using the Bruker software suite APEX3.[3] The final unit cell was obtained from the xyz centroids of 9824 reflections after integration. A multiscan absorption correction was applied, based on the intensities of symmetry-related reflections measured at different angular settings (SADABS). The structures were solved by direct methods using SHELXT.[4] and refinement of the structure was performed using SHLELXL.[5] The hydrogen atoms were generated by geometrical considerations, constrained to idealized geometries and allowed to ride on their carrier atoms with an isotropic displacement parameter related to the equivalent displacement parameter of their carrier atoms.

Synthesis methods

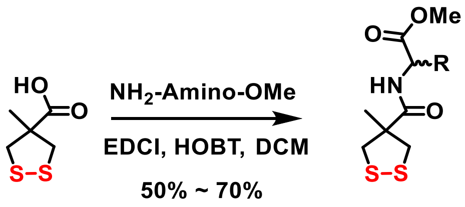

General method:

All the compounds were synthesized by a typical amidation reaction of carboxylic acids (MAA or AA) and deprotected amines. In a typical procedure, MAA or AA was dissolved in CH_2Cl_2 (10 mL; 100 mM) in a flask to obtain a homogeneous bright yellow solution. After cooling down to 0°C, coupling reagents (EDCI, 1.2 eq; HOBT, 1.2 eq) were slowly added under vigorous stirring. After pre-activation for 30 min, deprotected amines (R-NH_2, 1.5 eq) in CH_2Cl_2 or DMF (dependent on the solubility) were dropwisely added into the mixture. Process of the reaction was monitored by TLC and finished in 2 ~ 6 h. Then the reaction mixture was washed by 1 M HCl (aq) (50 mL × 3), H_2O (50 mL × 1), saturated aq. $NaHCO_3$ (50 mL × 3), and brine (50 mL × 1). The organic phase was dried by anhydrous Na_2SO_4, and purified by flash chromatography (SiO_2, CH_2Cl_2/CH_3OH = 200 : 1 to 50 : 1). The products were carefully collected by evaporating solvents under vacuum at low temperature (below 35°C) to avoid polymerization, affording yellow powders or crystals (yield = 30% ~ 60%), which can be stored in the fridge for several months.

AA-Gly: Light yellow crystals.

^{1}H NMR ($CDCl_3$, 400 MHz, 298 K, ppm): δ = 7.178 (s, 1H), 3.96 (d, J = 4.8 Hz, 2H), 3.66 (s, 3H), 3.27 (m, 5H), 1.99 (s, 3H), 1.41 (s, 3H).

^{13}C NMR ($CDCl_3$, 101 MHz, 298 K, ppm) δ = 172.0, 170.1, 52.4, 51.9, 42.7, 41.3.

HR-MS (ESI) (m/z): [M + H^+] Measured: 221.0175; Calculated: 221.0175

AA-L-Phe: Light yellow crystals.

^{1}H NMR ($CDCl_3$, 500 MHz, 298 K, ppm) δ 7.31 (m, 3H), 7.12 (d, J = 8.5 Hz, 2H), 6.28 (d, J = 7.0 Hz, 1H), 4.92 (m, 1H), 3.78 (s, 3H), 3.29 (m, 6H), 3.09 (m, 1H).

^{13}C NMR ($CDCl_3$, 101 MHz, 298 K, ppm): δ = 171.8, 171.2, 135.6, 129.1, 128.6, 127.3, 53.1, 52.5, 52.0, 42.8, 42.4, 37.6.

HR-MS (ESI) (m/z): [M + H^+] Measured: 312.0722; Calculated: 312.0723.

AA-dmGly: Light yellow solids.

^{1}H NMR ($CDCl_3$, 500 MHz, 298 K, ppm): δ = 6.40 (s, 1H), 3.73 (s, 3H), 3.35 (m, 4H), 3.17 (m, 1H), 1.54 (s, 6H).

^{13}C NMR ($CDCl_3$, 101 MHz, 298 K, ppm): δ = 174.8, 171.1, 56.8, 52.7, 52.4, 42.8, 24.7, 24.7.

HR-MS (ESI) (m/z): [M + H^+] Measured: 250.0563; Calculated: 250.0566.

MAA-D-Ben: Colorless needles.

^{1}H NMR ($CDCl_3$, 400 MHz, 293 K, ppm) δ 7.35 (m, 5H), 7.02 (d, J = 7.2 Hz, 1H), 5.52 (d, J = 6.8 Hz, 1H), 3.72 (s, 3H), 3.59 (d, J = 12.0 Hz, 1H), 3.48 (d, J = 12.0 Hz, 1H), 3.01 (dd, J_1 = 5.2 Hz, J_2 = 4.8 Hz, 2H), 1.45 (s, 3H).

^{13}C NMR ($CDCl_3$, 101 MHz, 298 K, ppm) δ = 174.2, 171.2, 136.0, 129.0, 128.6, 127.1, 56.8, 56.3, 52.8, 50.1, 50.1, 23.7.

HR-MS (ESI) (m/z): [M + Na^+] Measured: 334.0546; Calculated: 334.0542.

MAA-L-Ala-OBu: Yellow powder.

^{1}H NMR ($CDCl_3$, 500 MHz, 298 K, ppm): δ = 6.51 (s, 1H), 4.42 (m, 1H), 3.54 (dd, J_1 = 12.0 Hz, J_2 = 12.0 Hz, 2H), 2.98 (dd, J_1 = 12.0 Hz, J_2 = 12.0 Hz, 2H), 1.48 (s, 9H), 1.43 (s, 3H), 1.37 (d, J = 7.0 Hz, 3H).

^{13}C NMR ($CDCl_3$, 101 MHz, 298 K, ppm): δ = 174.0, 172.0, 82.1, 56.4, 50.1, 50.0, 48.9, 27.9, 23.8, 18.4.

HR-MS (ESI) (m/z): [M + Na^+] Measured: 314.0853; Calculated: 314.0855.

MAA-R-1-NP: Yellow crystals.

^{1}H NMR ($CDCl_3$, 500 MHz, 298 K, ppm): δ = 8.04 (d, J = 8.0 Hz, 2H), 7.88 (d, J = 7.5 Hz, 1H), 7.82 (d, J = 8.0 Hz, 1H), 7.54 (m, 4H), 6.28 (s, 1H), 5.92 (m, 1H), 3.52 (dd, J_1 = 12.0 Hz, J_2 = 12.0 Hz, 2H), 2.97 (dd, J_1 = 12.0 Hz, J_2 = 12.0 Hz, 2H), 1.67 (d, J = 7.0 Hz, 3H), 1.42 (s, 3H).

^{13}C NMR ($CDCl_3$, 101 MHz, 298 K, ppm): δ = 173.4, 138.1, 133.9, 131.0, 128.9, 128.4, 126.5, 125.9, 125.2, 123.2, 122.5, 56.6, 50.1, 49.6, 45.2, 23.8, 20.7.

HR-MS (ESI) (m/z): [M + H^+] Measured: 318.0978; Calculated: 318.0981.

MAA-R-2-NP: Yellow crystals.

^{1}H NMR ($CDCl_3$, 400 MHz, 293 K, ppm): δ = 7.82 (m, 3H), 7.75 (s, 1H), 7.48 (m, 2H), 7.42 (d, J = 8.8 Hz, 1H), 6.41 (d, J = 7.6 Hz, 1H), 5.27 (m, 1H), 3.53 (dd, J_1 = 12.0 Hz, J_2 = 11.6 Hz, 2H), 3.03 (dd, J_1 = 11.6 Hz, J_2 = 12.0 Hz, 2H), 1.60 (d, J = 7.2 Hz, 3H), 1.68 (d, J = 12 Hz, 2H), 1.45 (s, 3H).

^{13}C NMR ($CDCl_3$, 101 MHz, 298 K, ppm): δ = 173.7, 140.3, 133.3, 132.7, 128.6, 127.9, 127.6, 126.3, 125.9, 124.5, 124.4, 56.6, 50.0, 49.9, 49.3, 23.8, 21.7.

HR-MS (ESI) (m/z): [M + H^+] Measured: 318.0979; Calculated: 318.0981.

MAA-L-Ala-L-Ala: Yellow powder.

^{1}H NMR ($CDCl_3$, 400 MHz, 298 K, ppm) δ 6.71 (m, 2H), 4.53 (m, 2H), 3.75 (s, 3H), 3.56 (dd, J_1 = 11.6 Hz, J_2 = 11.6 Hz, 2H), 3.01 (dd, J_1 = 11.6 Hz, J_2 = 11.6 Hz, 2H), 1.46 (s, 3H), 1.42 (d, J = 7.2 Hz, 6H).

^{13}C NMR ($CDCl_3$, 101 MHz, 298 K, ppm): δ = 173.7, 140.3, 133.3, 132.7, 128.6, 127.9, 127.6, 126.3, 125.9, 124.5, 124.4, 56.6, 50.0, 49.9, 49.3, 23.8, 21.7.

HR-MS (ESI) (m/z): [M + Na^+] Measured: 343.0755; Calculated: 343.0757.

MAA-L-Leu-L-Leu: Yellow powder.

^{1}H NMR ($CDCl_3$, 500 MHz, 298 K, ppm) δ 6.64 (d, J = 8.0 Hz, 1H), 6.57 (d, J = 8.0 Hz, 1H), 4.54 (m, 2H), 3.71 (s, 3H), 3.53 (dd, J_1 = 12.0 Hz, J_2 = 12.0 Hz, 2H), 3.00 (dd, J_1 = 11.5 Hz, J_2 = 11.5 Hz, 2H), 1.60 (m, 6H), 1.44 (s, 3H), 0.94 (m, 12H).

^{13}C NMR ($CDCl_3$, 101 MHz, 298 K, ppm): δ = 174.6, 173.0, 171.7, 56.9, 52.2, 52.0, 50.8, 49.3, 49.2, 41.2, 40.8, 24.9, 24.8, 23.7, 22.9, 22.7, 22.1, 21.8.

HR-MS (ESI) (m/z): [M + H^+] Measured: 405.1871; Calculated: 405.1876.

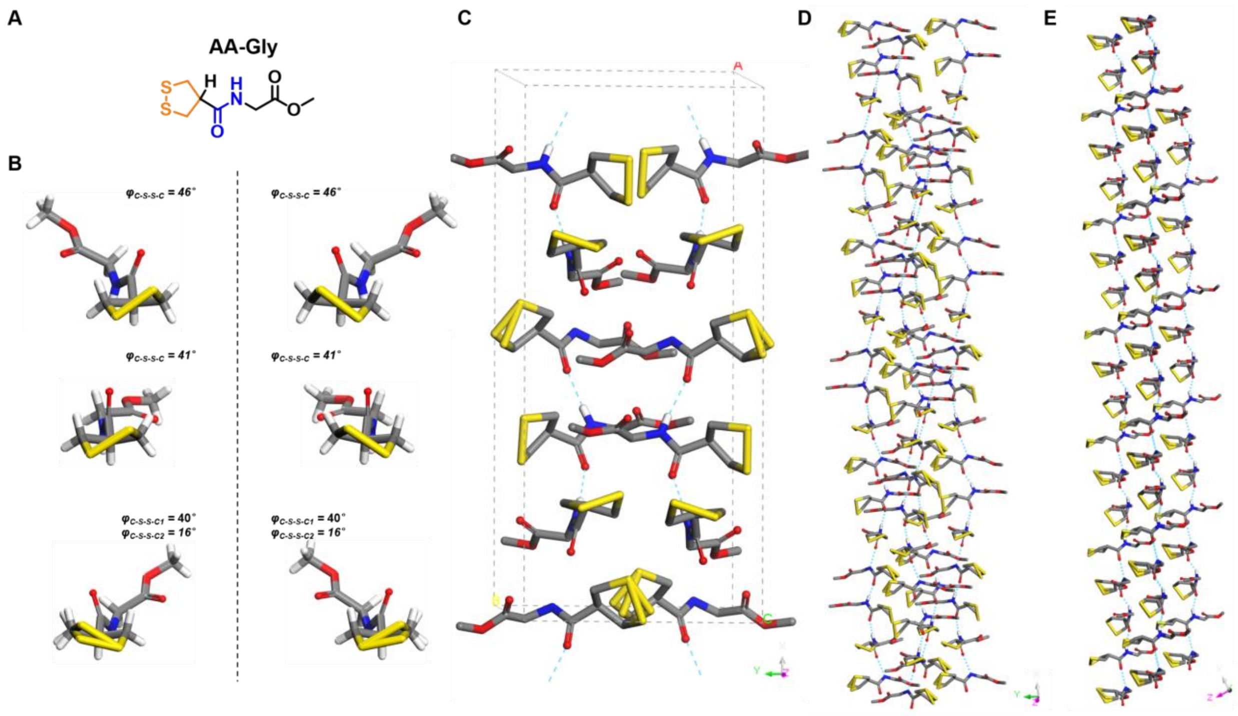

Fig. S1.

X-ray single-crystal structure of AA-Gly. A) Molecular structure; B) Representative molecular geometries extracted from the assemblies; C) Unit cell of the crystal; D) Supramolecular packing architecture along H-bonding direction.

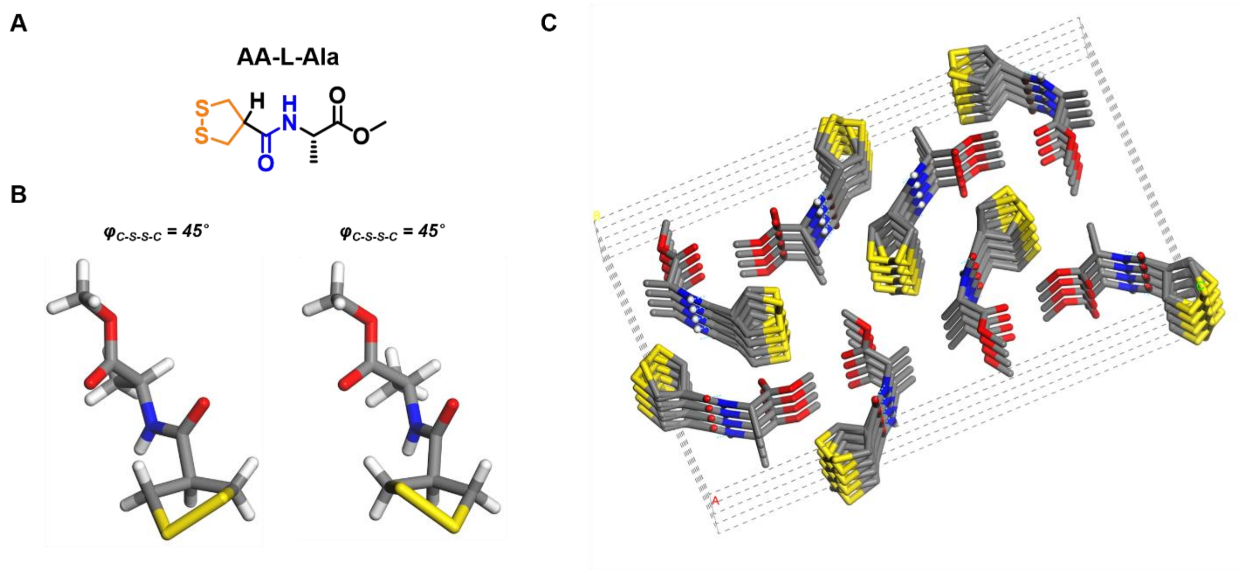

Fig. S2.

X-ray single-crystal structure of AA-L-Ala. A) Molecular structure; B) Representative molecular geometries extracted from the assemblies; C) Supramolecular packing architecture.

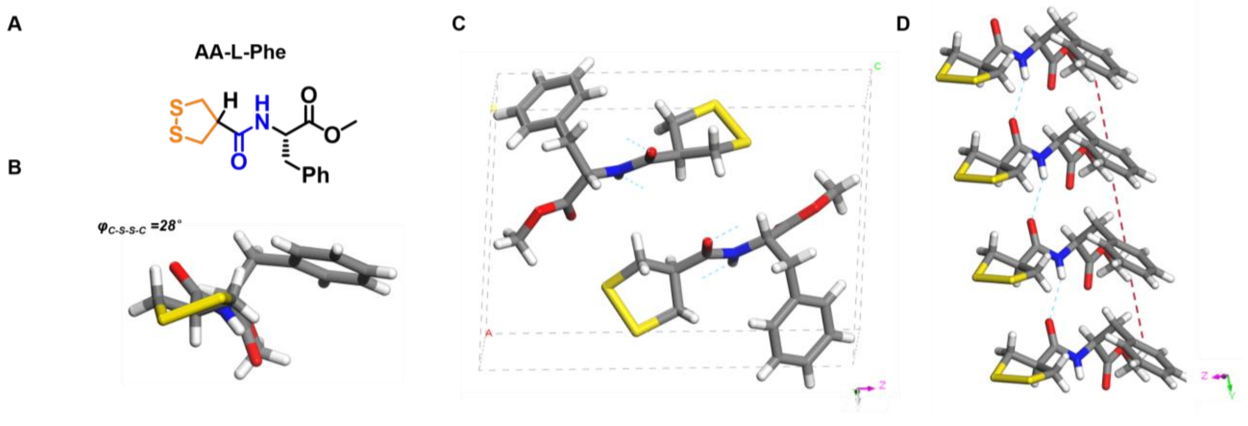

Fig. S3.

X-ray single-crystal structure of AA-L-Phe. A) Molecular structure; B) Representative molecular geometries extracted from the assemblies; C) Unit cell; D) Supramolecular packing architecture. The red line represents the existing π-π stacking interactions.

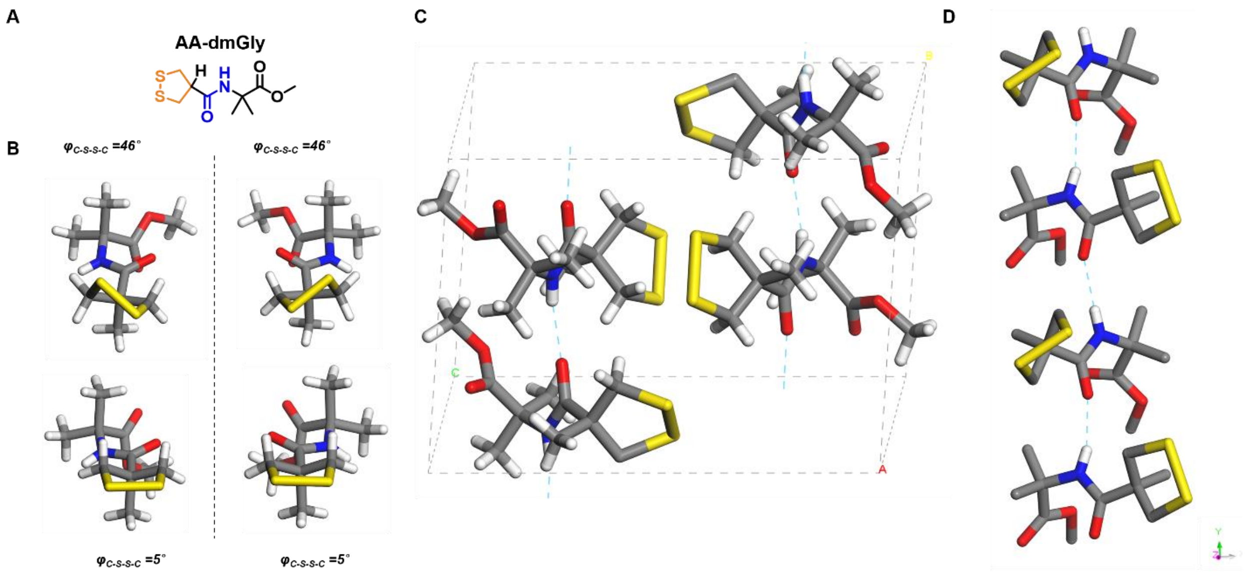

Fig. S4.

X-ray single-crystal structure of AA-dmGly. A) Molecular structure; B) Representative molecular geometries extracted from the assemblies; C) Unit cell; D) Supramolecular packing architecture along H-bonding direction.

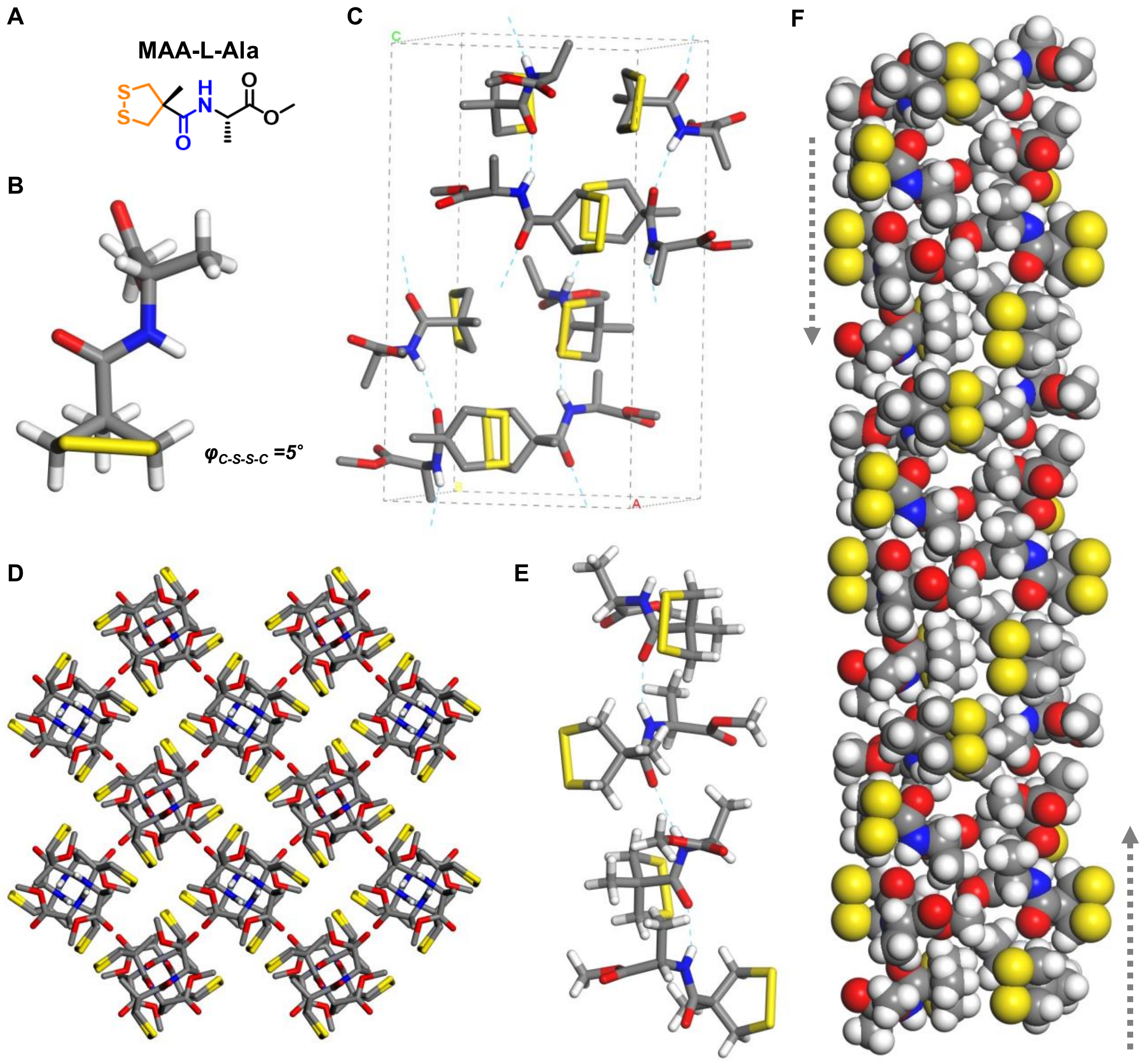

Fig. S5.

X-ray single-crystal structure of MAA-L-Ala. A) Molecular structure; B) Representative molecular geometries extracted from the assemblies; C) Unit cell; D-F) Supramolecular packing architecture.

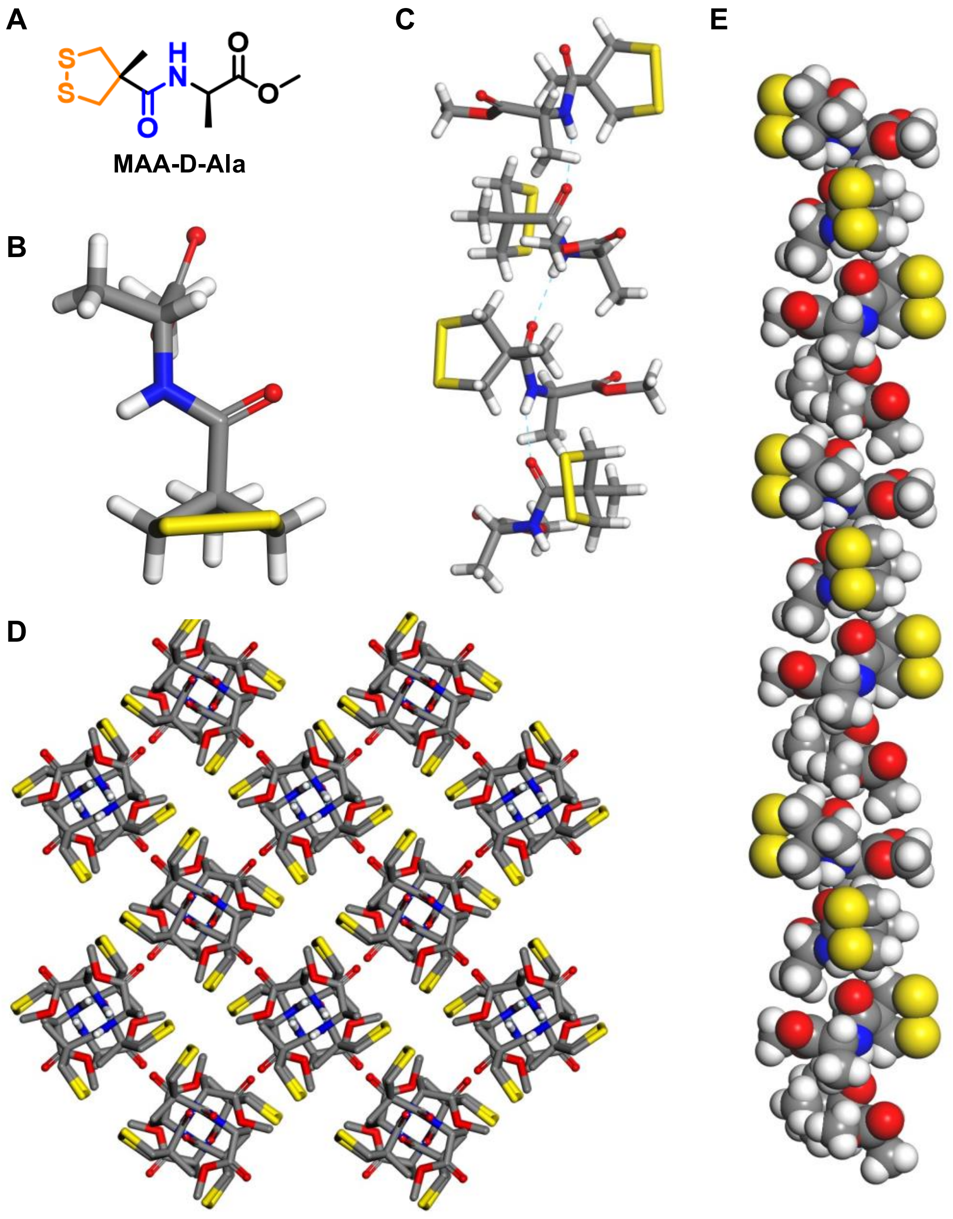

Fig. S6.

X-ray single-crystal structure of MAA-D-Ala. A) Molecular structure; B) Representative molecular geometries extracted from the assemblies; C-E) Supramolecular packing architecture.

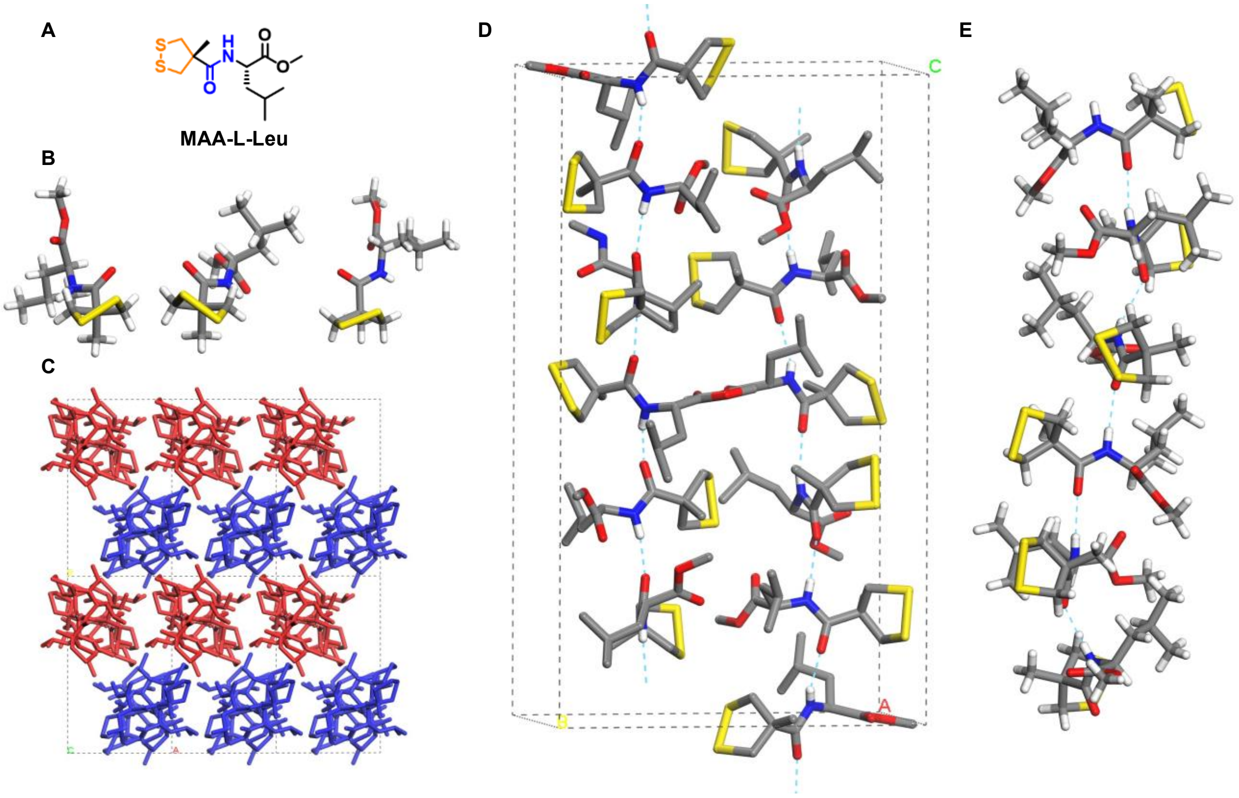

Fig. S7.

X-ray single-crystal structure of MAA-L-Leu. A) Molecular structure; B) Representative molecular geometries extracted from the assemblies; C-E) Supramolecular packing architecture.

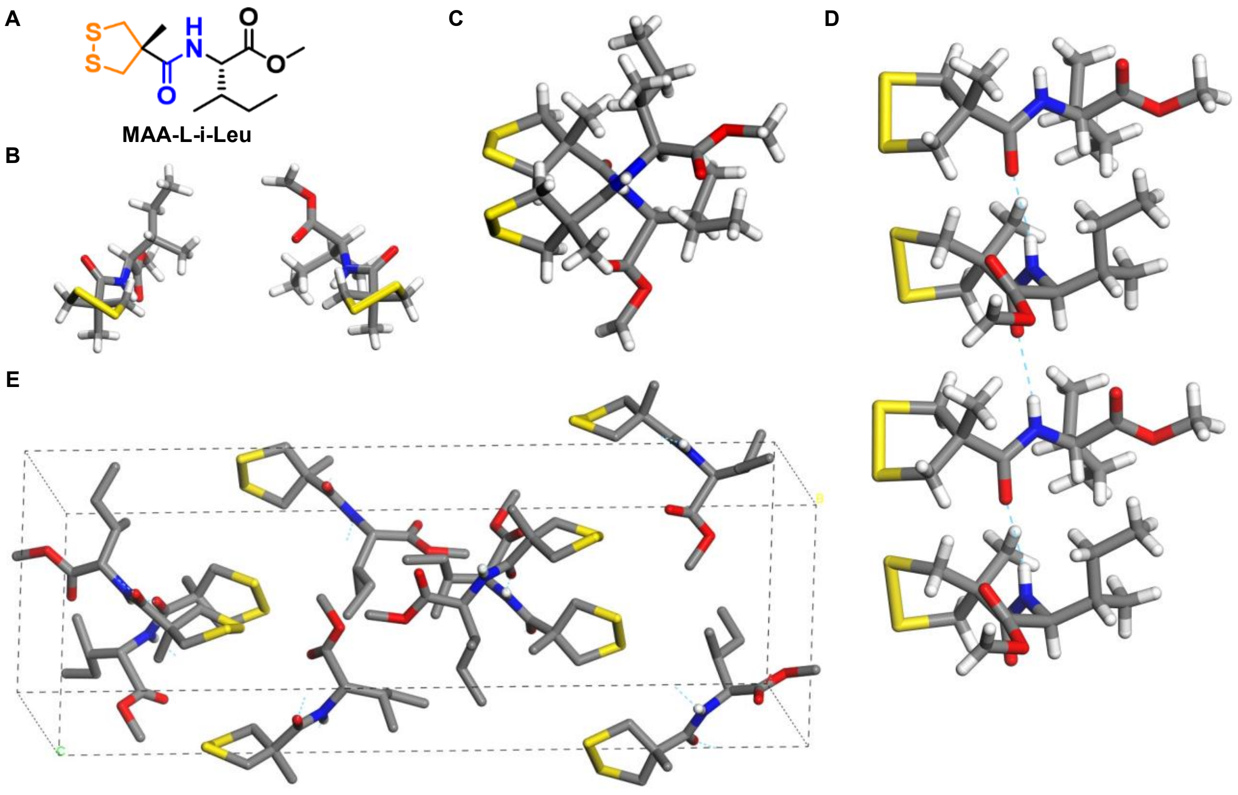

Fig. S8.

X-ray single-crystal structure of MAA-L-i-Leu. A) Molecular structure; B) Representative molecular geometries extracted from the assemblies; C-E) Supramolecular packing architecture.

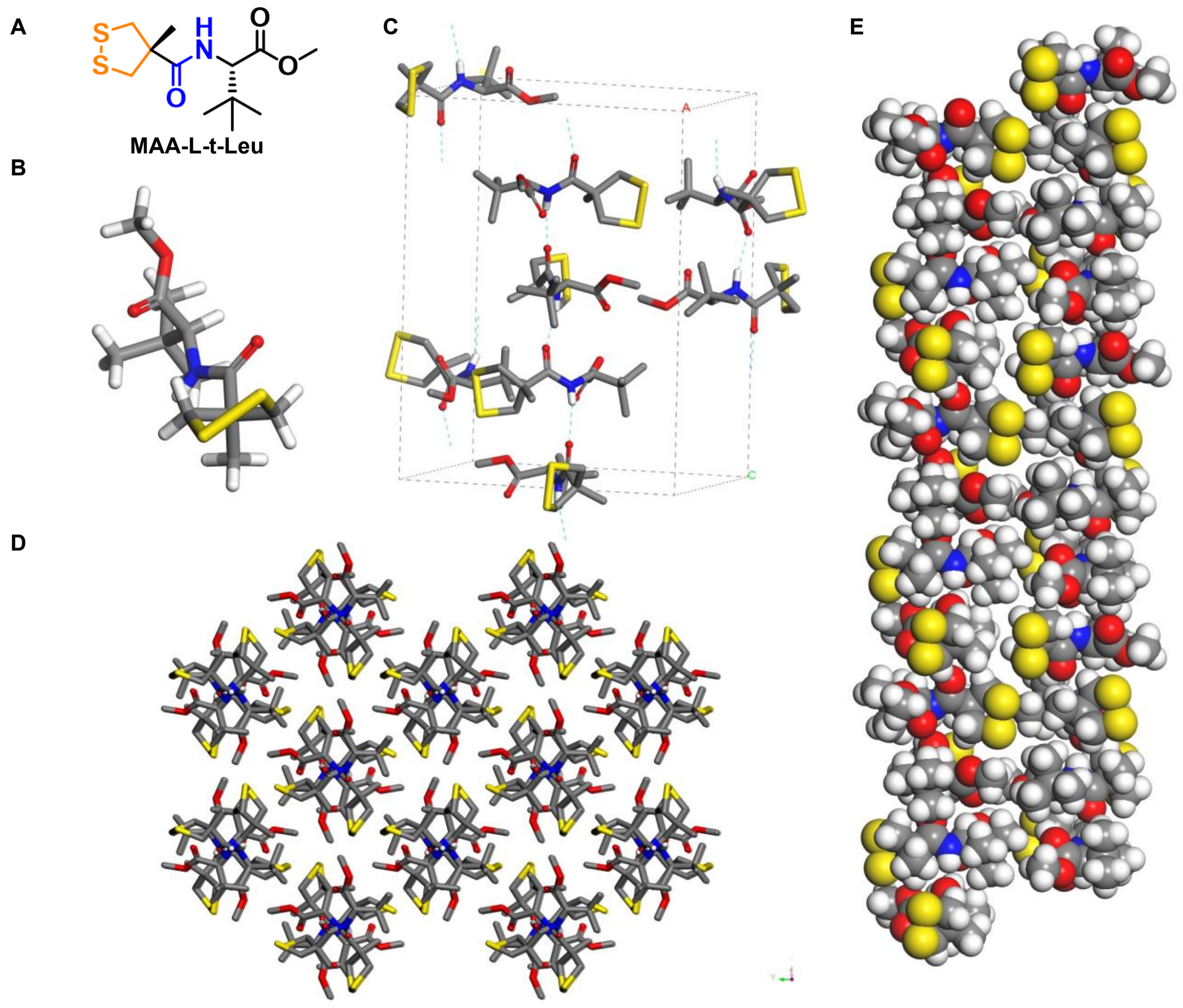

Fig. S9.

X-ray single-crystal structure of MAA-L-t-Leu. A) Molecular structure; B) Representative molecular geometries extracted from the assemblies; C-E) Supramolecular packing architecture.

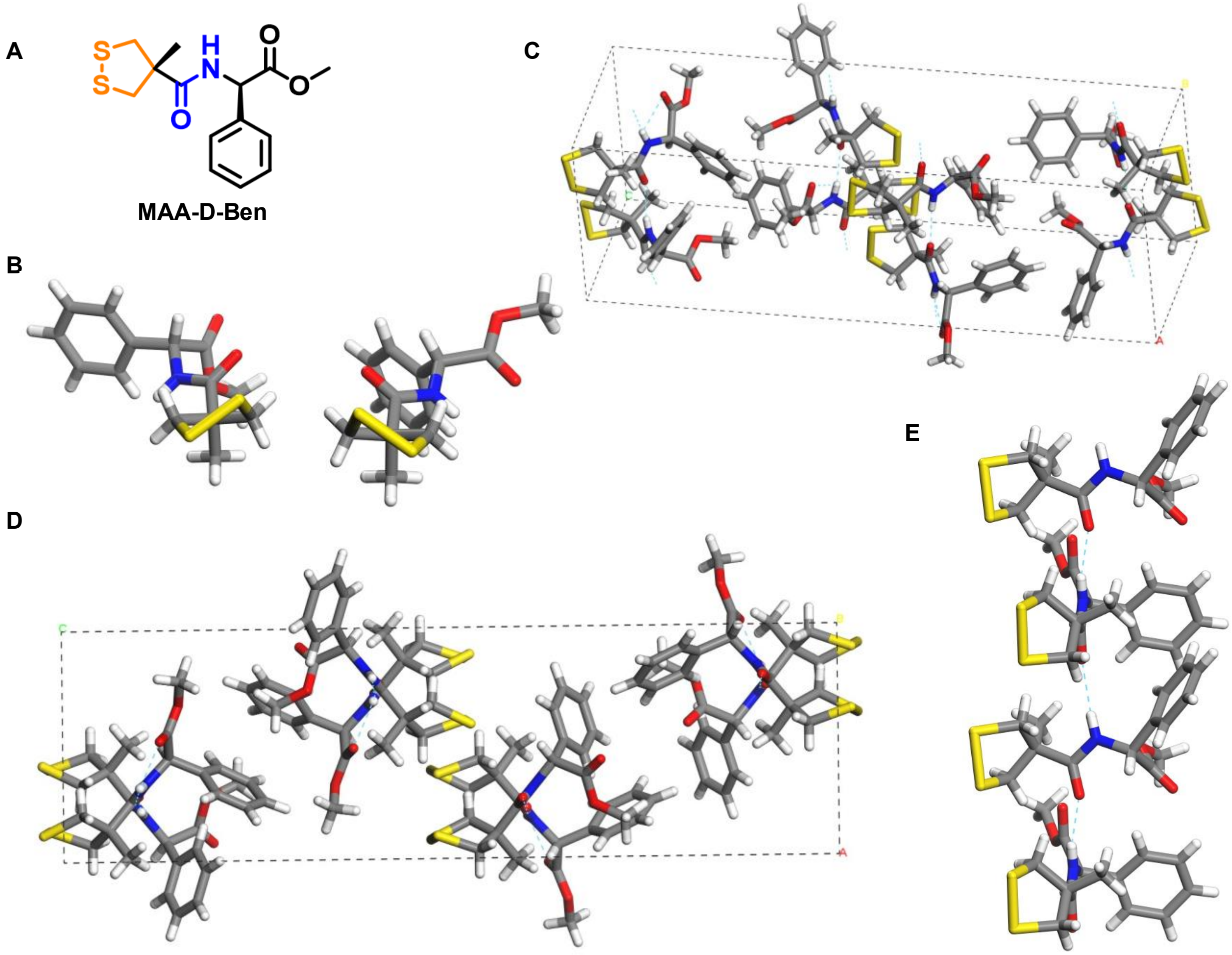

Fig. S10.

X-ray single-crystal structure of MAA-D-Ben. A) Molecular structure; B) Representative molecular geometries extracted from the assemblies; C-E) Supramolecular packing architecture.

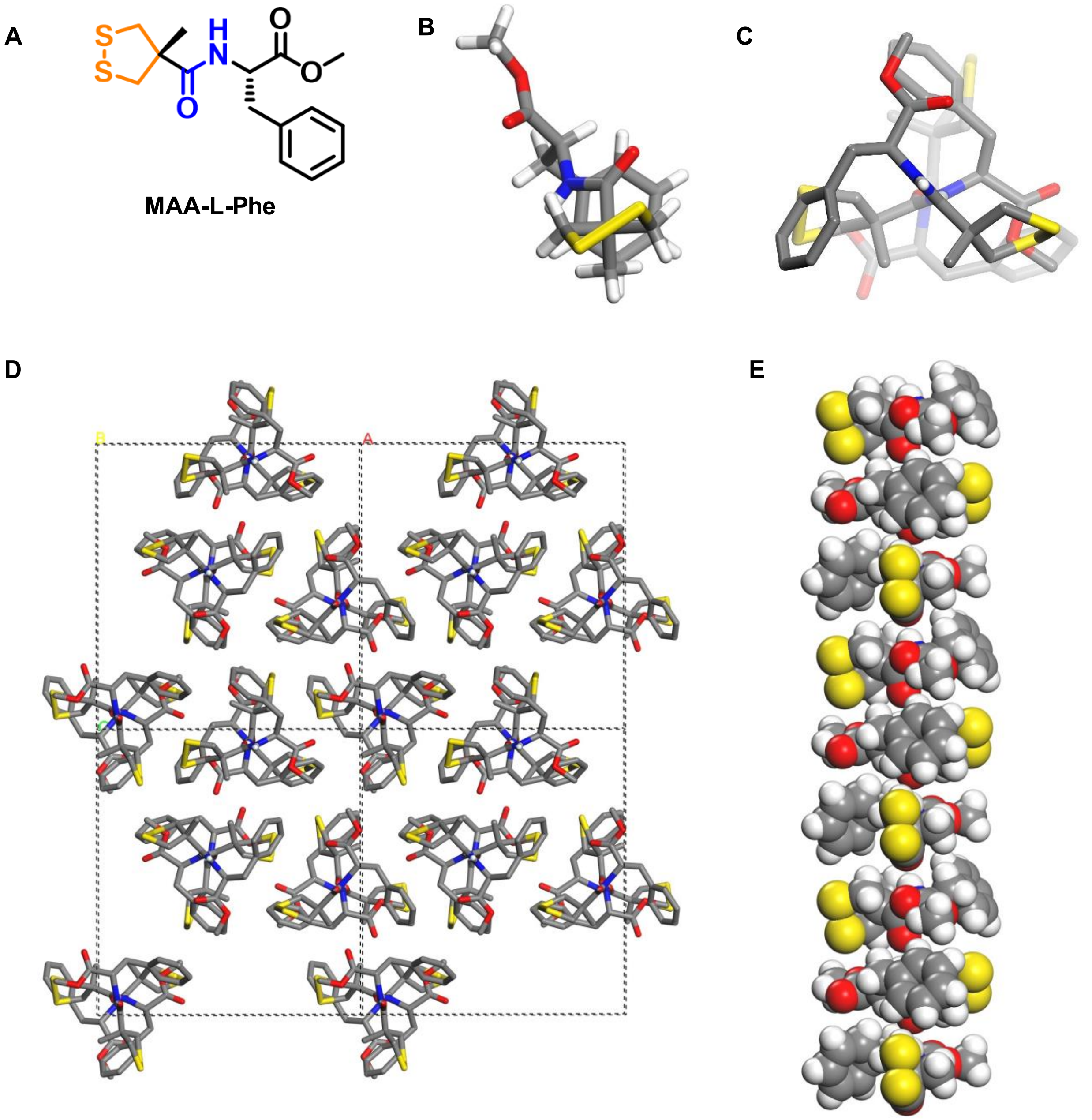

Fig. S11.

X-ray single-crystal structure of MAA-L-Phe. A) Molecular structure; B) Representative molecular geometries extracted from the assemblies; C-E) Supramolecular packing architecture.

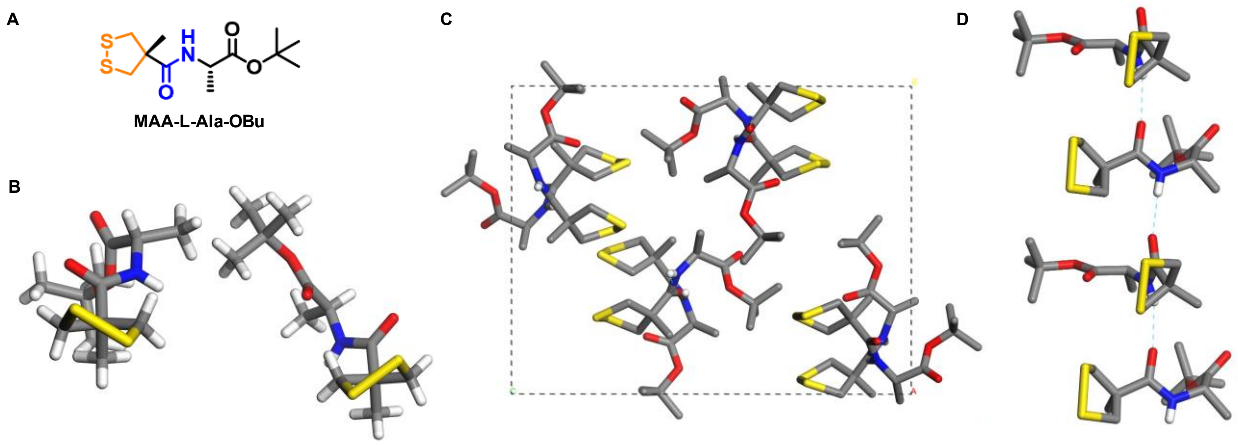

Fig. S12.

X-ray single-crystal structure of MAA-L-Ala-OBu. A) Molecular structure; B) Representative molecular geometries extracted from the assemblies; C,D) Supramolecular packing architecture.

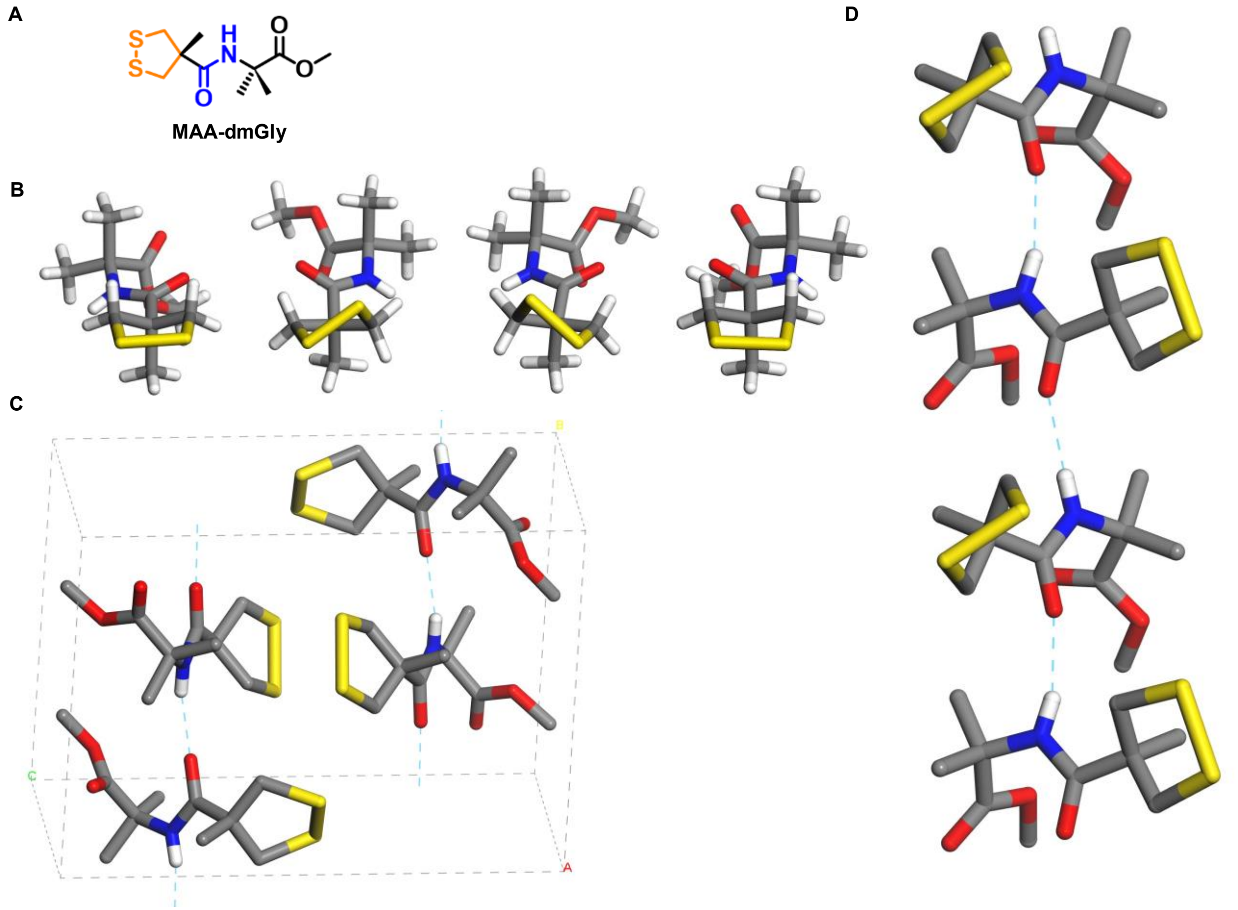

Fig. S13.

X-ray single-crystal structure of MAA-dm-Gly. A) Molecular structure; B) Representative molecular geometries extracted from the assemblies; C,D) Supramolecular packing architecture.

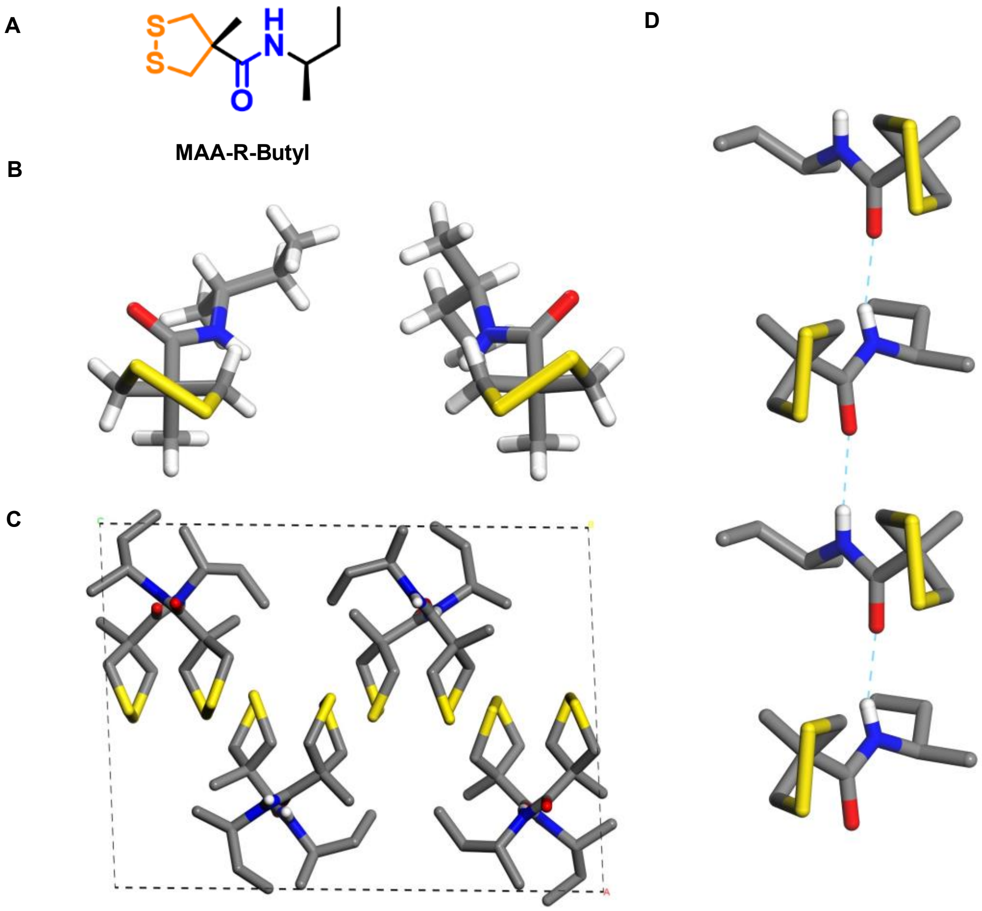

Fig. S14.

X-ray single-crystal structure of MAA-R-Butyl. A) Molecular structure; B) Representative molecular geometries extracted from the assemblies; C,D) Supramolecular packing architecture.

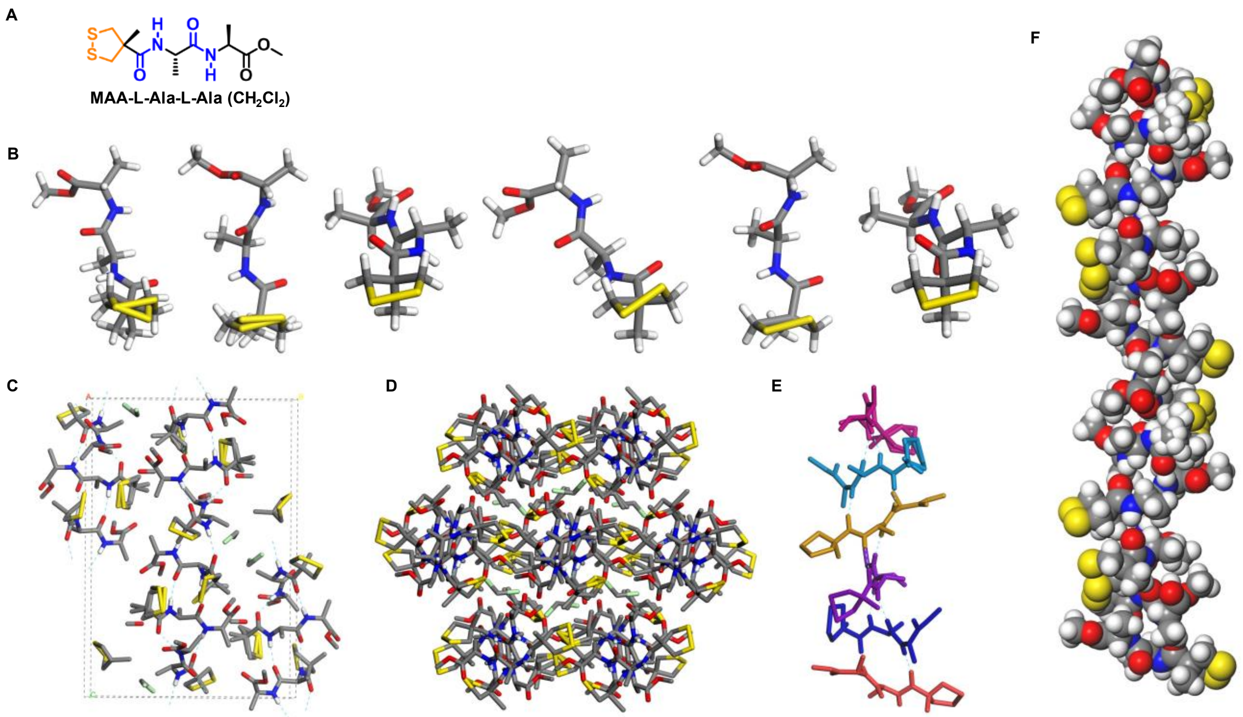

Fig. S15.

X-ray single-crystal structure of MAA-L-Ala-L-Ala (CH_2Cl_2). A) Molecular structure; B) Representative molecular geometries extracted from the assemblies; C-F) Supramolecular packing architecture.

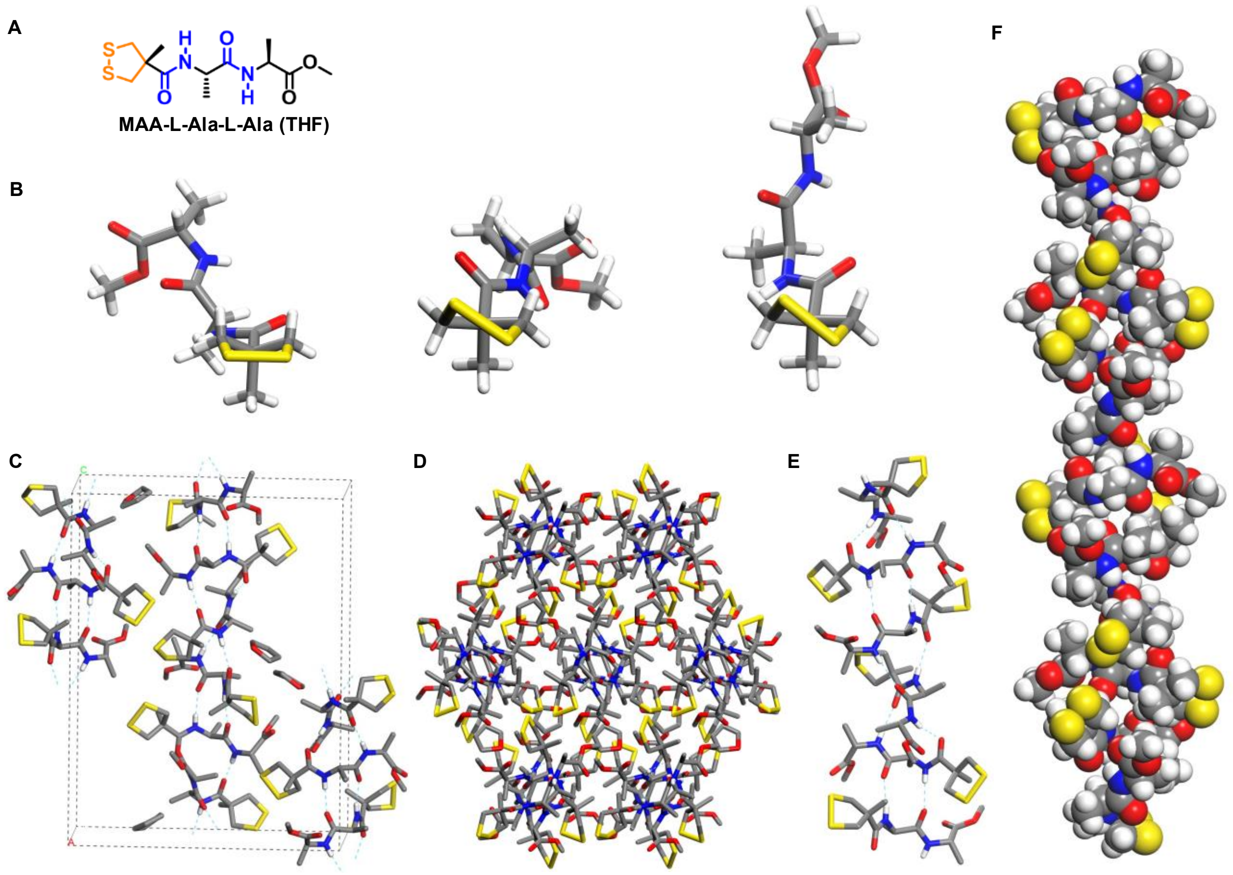

Fig. S16.

X-ray single-crystal structure of MAA-L-Ala-L-Ala (THF). A) Molecular structure; B) Representative molecular geometries extracted from the assemblies; C-F) Supramolecular packing architecture.

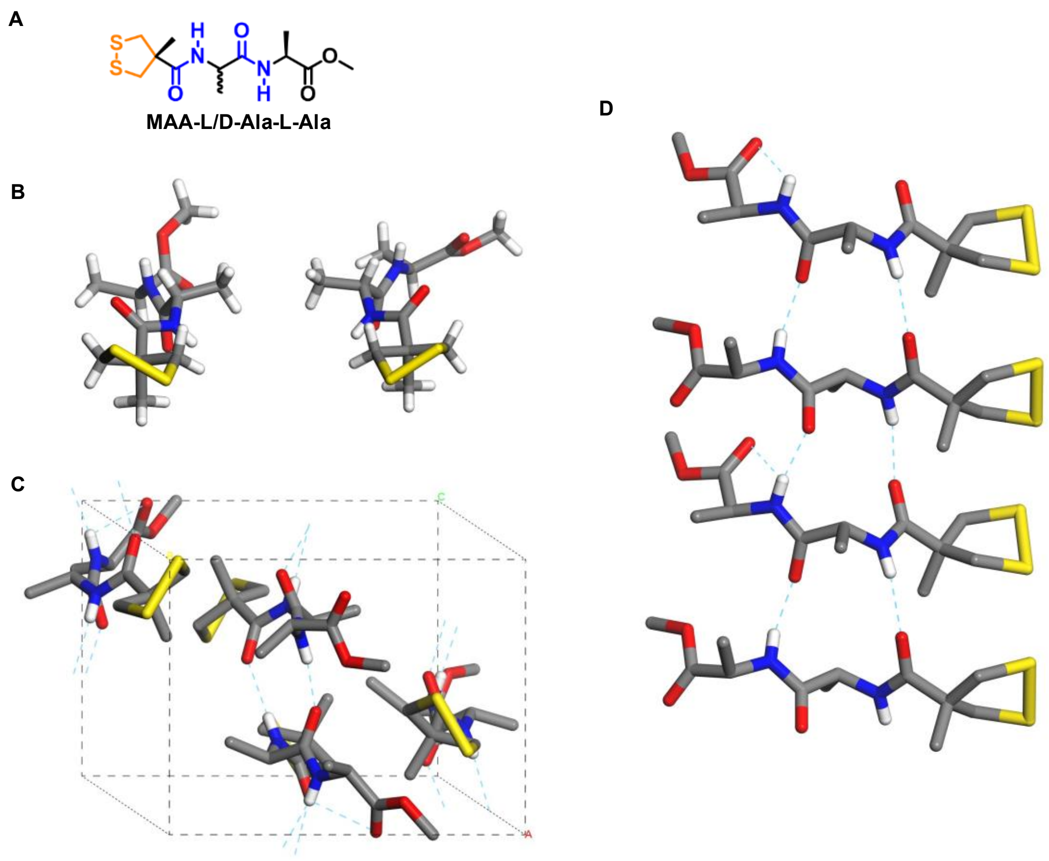

Fig. S17.

X-ray single-crystal structure of MAA-DL-Ala-L-Ala. A) Molecular structure; B) Representative molecular geometries extracted from the assemblies; C-D) Supramolecular packing architecture.

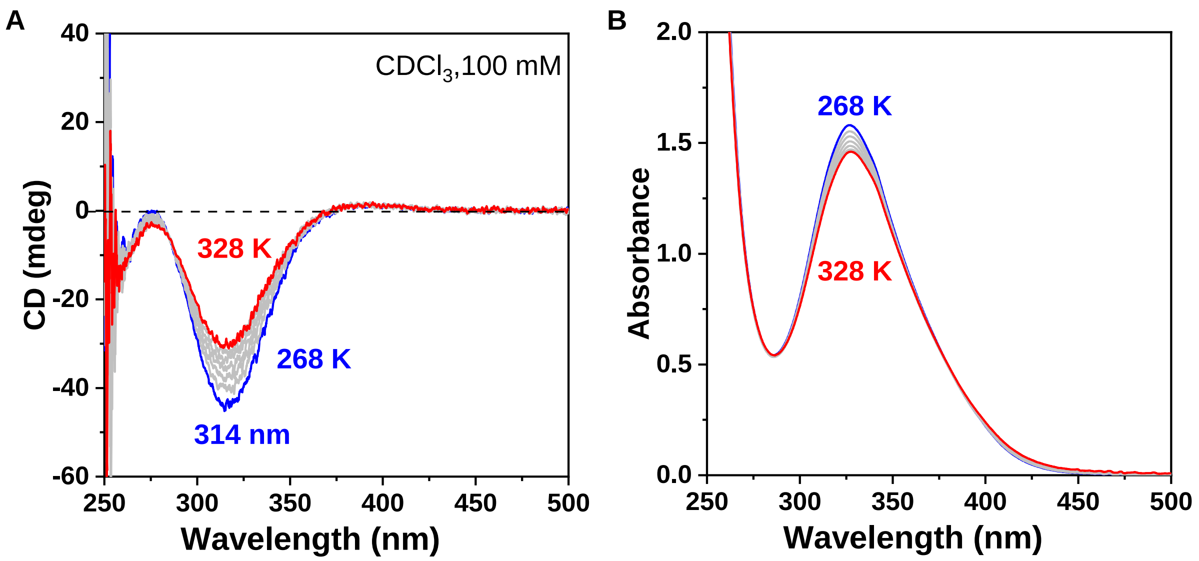

Fig. S18.

CD and UV-Vis spectra of MAA-L-Leu-L-Leu in concentrated solution ($CDCl_3$, 100 mM, optical path = 1 mm). The negative band at 314 nm indicated M-chiral disulfide bonds, which were consistent with the observations in solid states, indicating possibly remained helical supramolecular structures in concentrated solution.

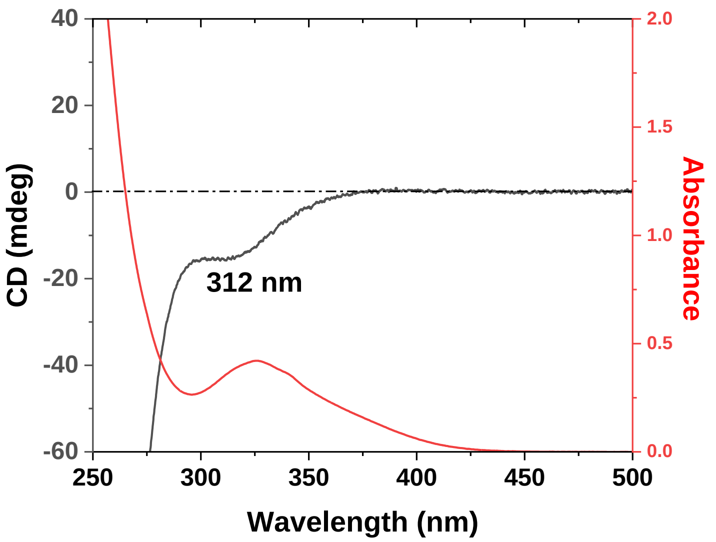

Fig. S19.

CD and UV-Vis spectra of MAA-L-Leu-L-Leu in diluted solution ($CDCl_3$, 2 mM, optical path = 10 mm). Compared with Fig. S21, the different CD band (250 ~ 300 nm) indicated the lack of β-sheet H-bond in diluted solutions. The disulfide bond showed a negative CD band at 312 nm due to the intramolecular chirality transfer induced by S-S···H-N H-bond.[6]

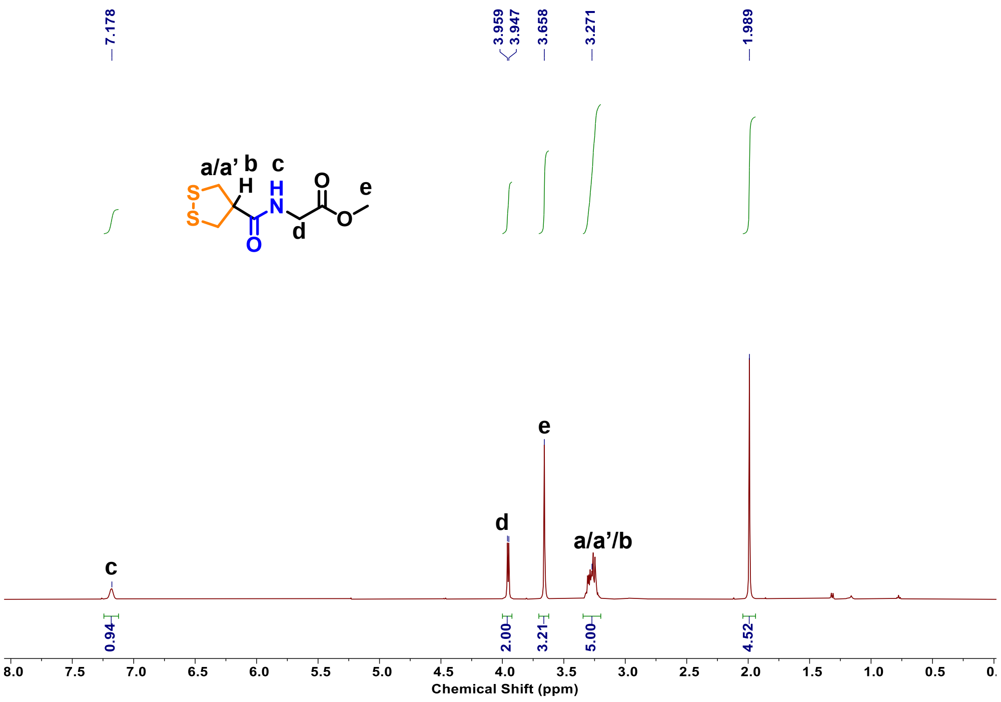

Fig. S20.

^{1}H NMR spectrum of AA-Gly in $CDCl_3$ (400 MHz, 298K).

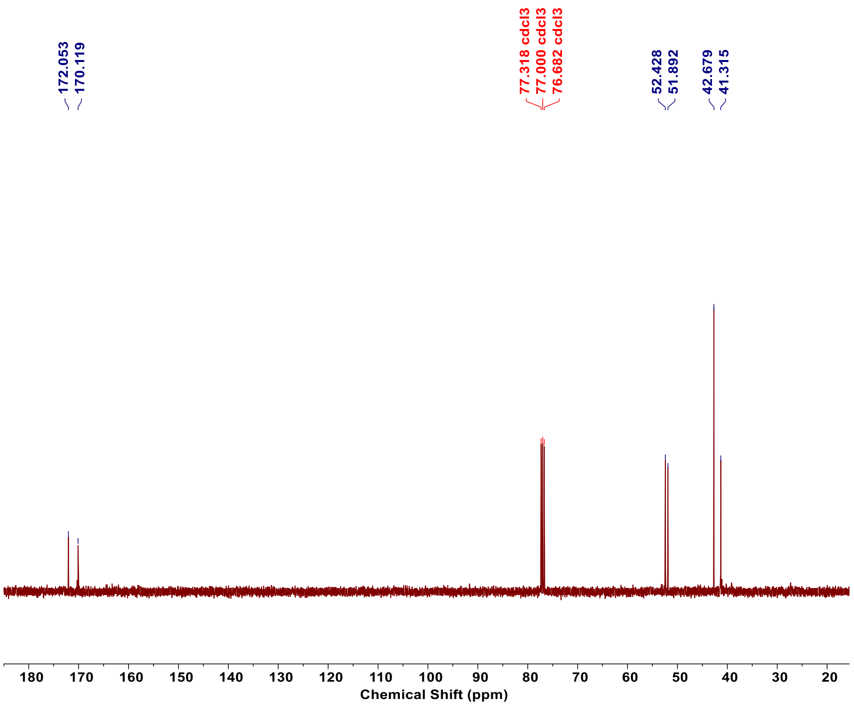

Fig. S21.

^{13}C NMR spectrum of AA-Gly in $CDCl_3$ (101 MHz, 298K).

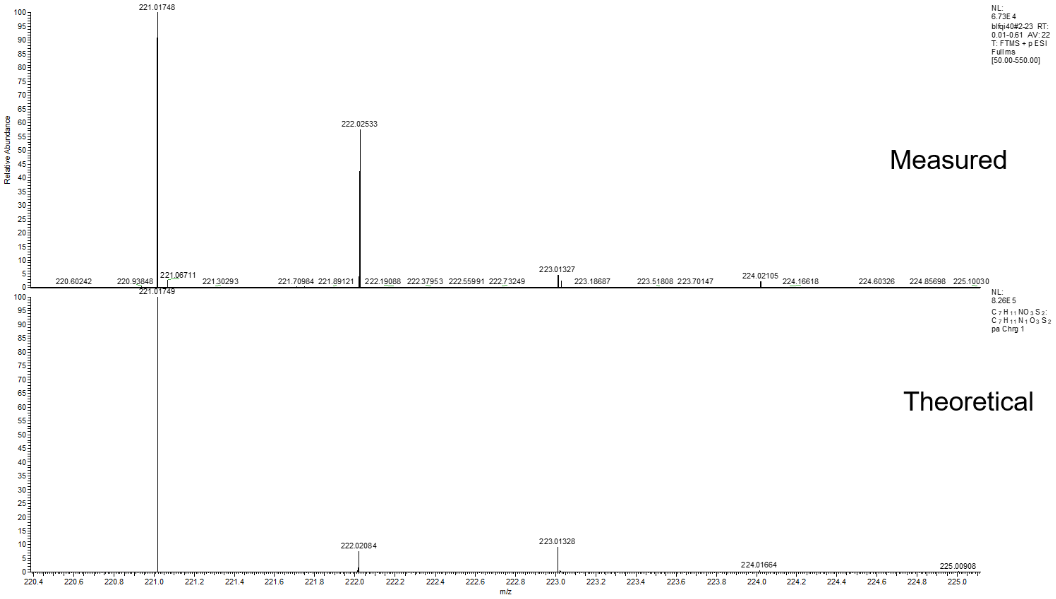

Fig. S22.

HR-MS of AA-Gly (Measured: 221.0175; Calculated: 221.0175).

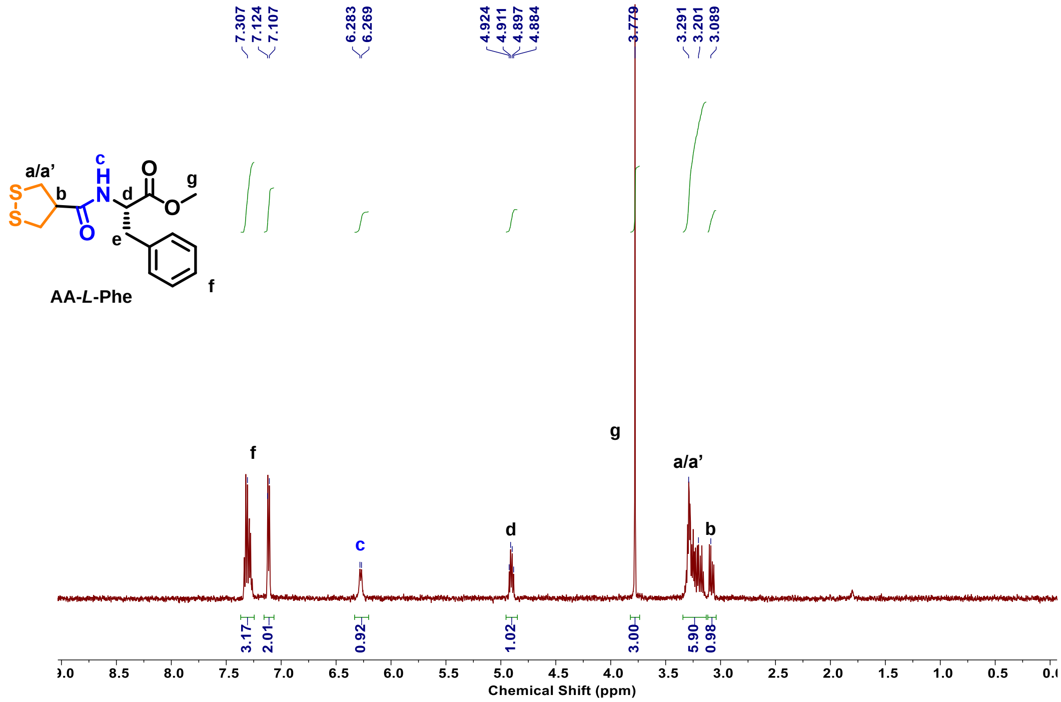

Fig. S23.

^{1}H NMR spectrum of AA-L-Phe in $CDCl_3$ (500 MHz, 298K).

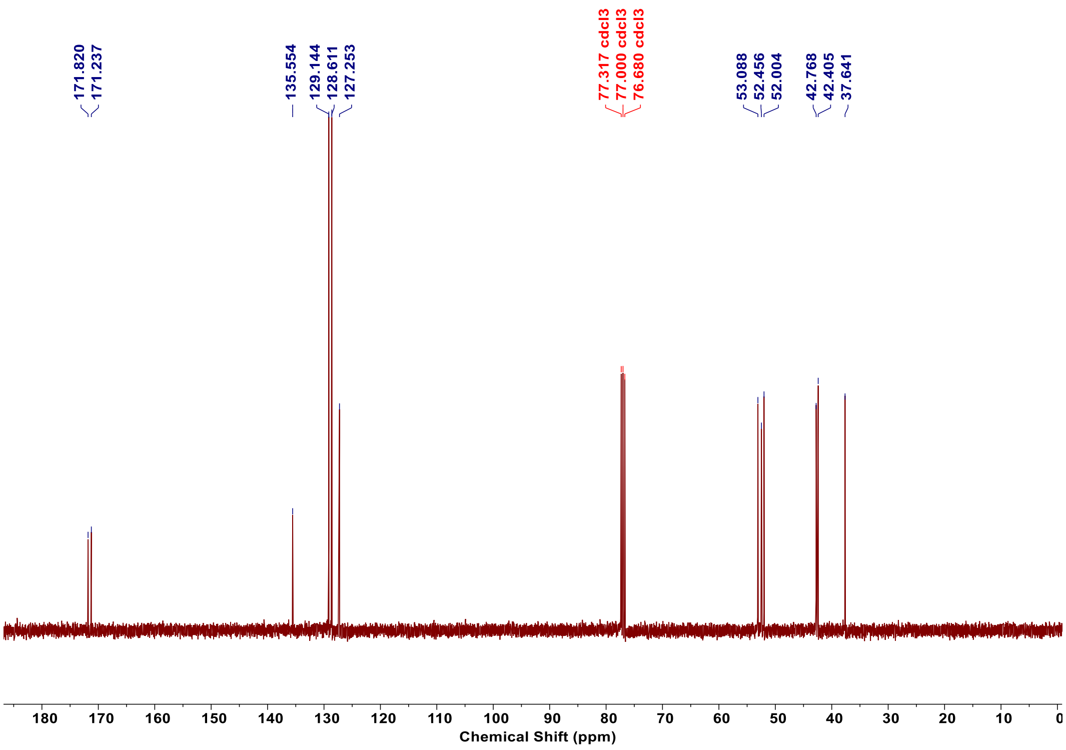

Fig. S24.

^{13}C NMR spectrum of AA-L-Phe in $CDCl_3$ (101 MHz, 298K).

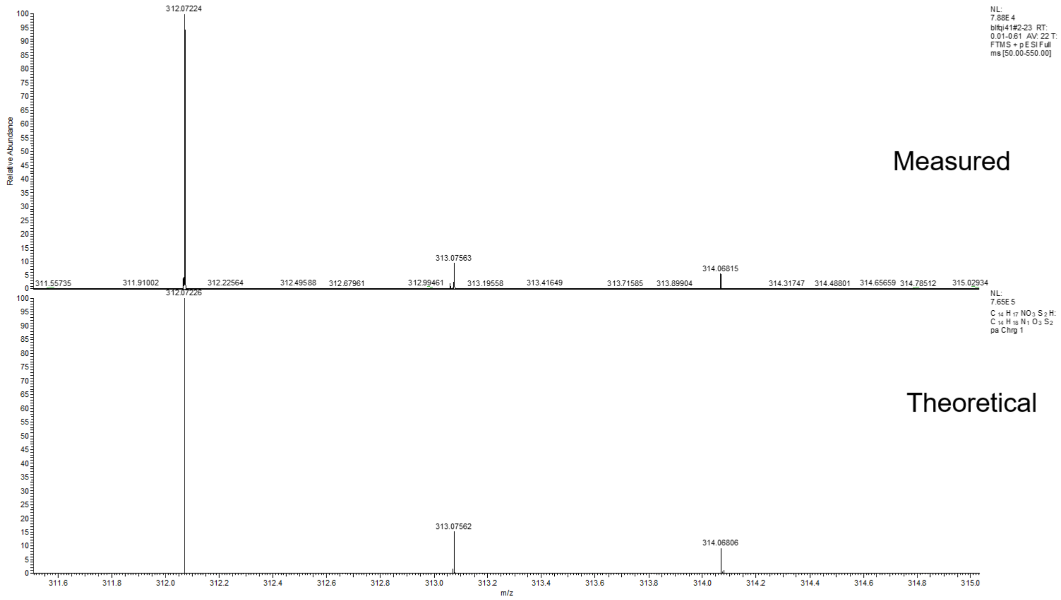

Fig. S25.

HR-MS of AA-L-Phe (Measured: 312.0722; Calculated: 312.0723).

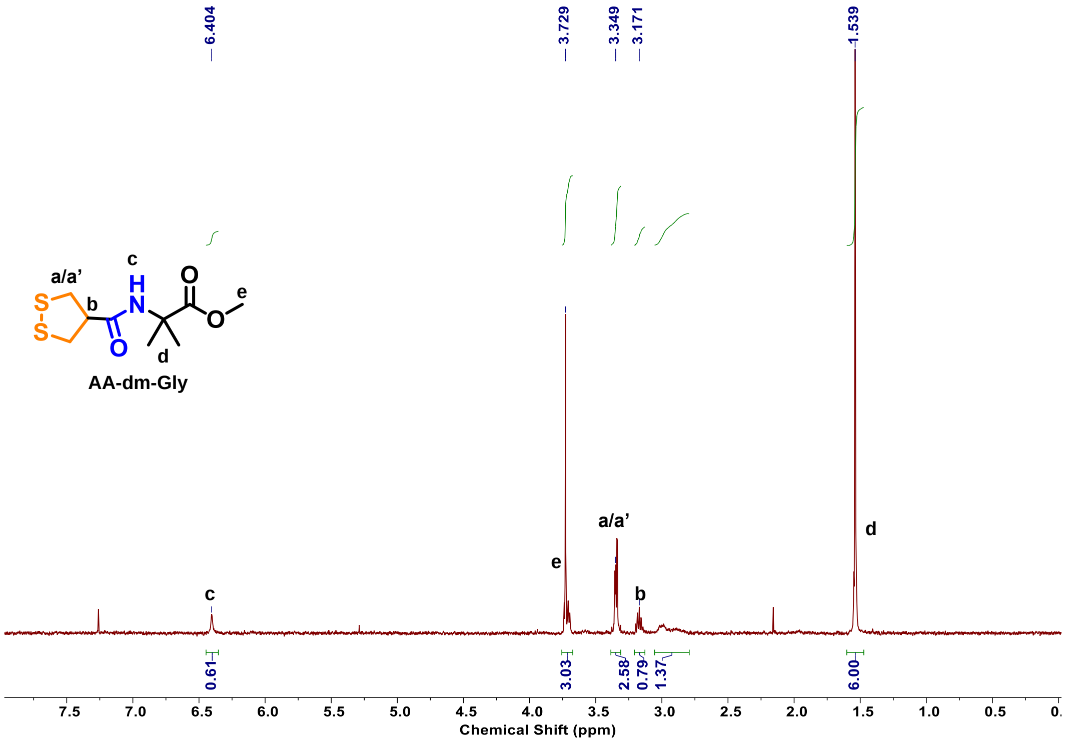

Fig. S26.

^{1}H NMR spectrum of AA-dmGly in $CDCl_3$ (500 MHz, 298K). The broad peaks at around 3 ppm are attributed to the polymers.

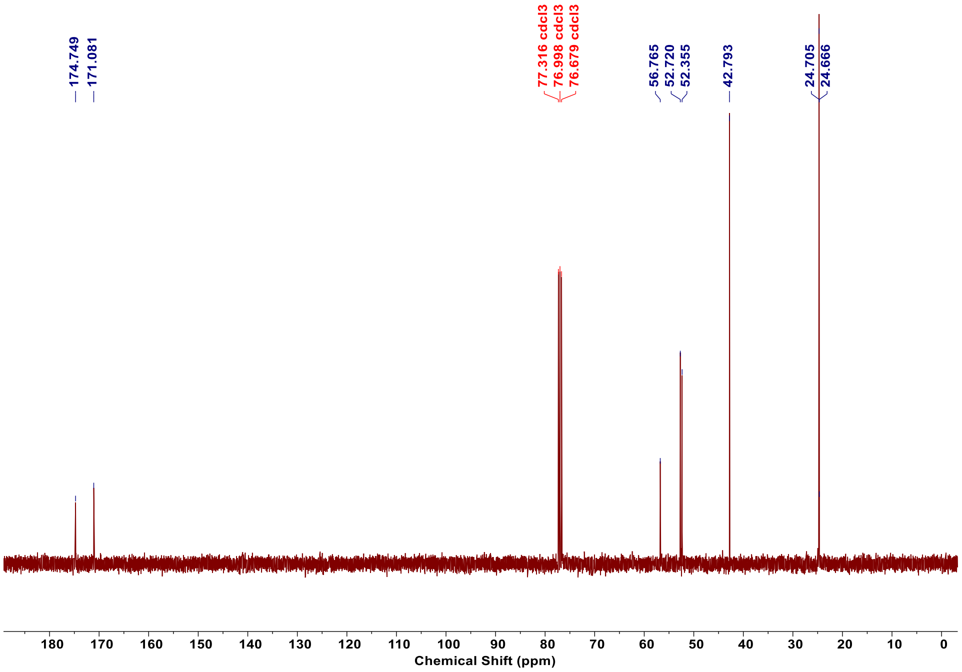

Fig. S27.

^{13}C NMR spectrum of AA-dmGly in $CDCl_3$ (101 MHz, 298K).

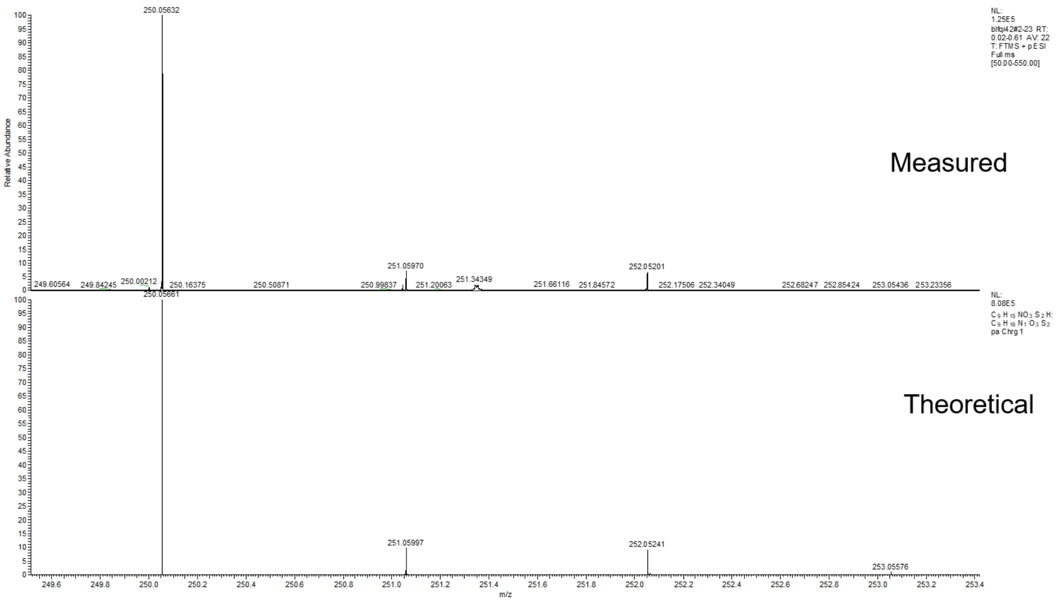

Fig. S28.

HR-MS of AA-dmGly (Measured: 250.0563; Calculated: 250.0566).

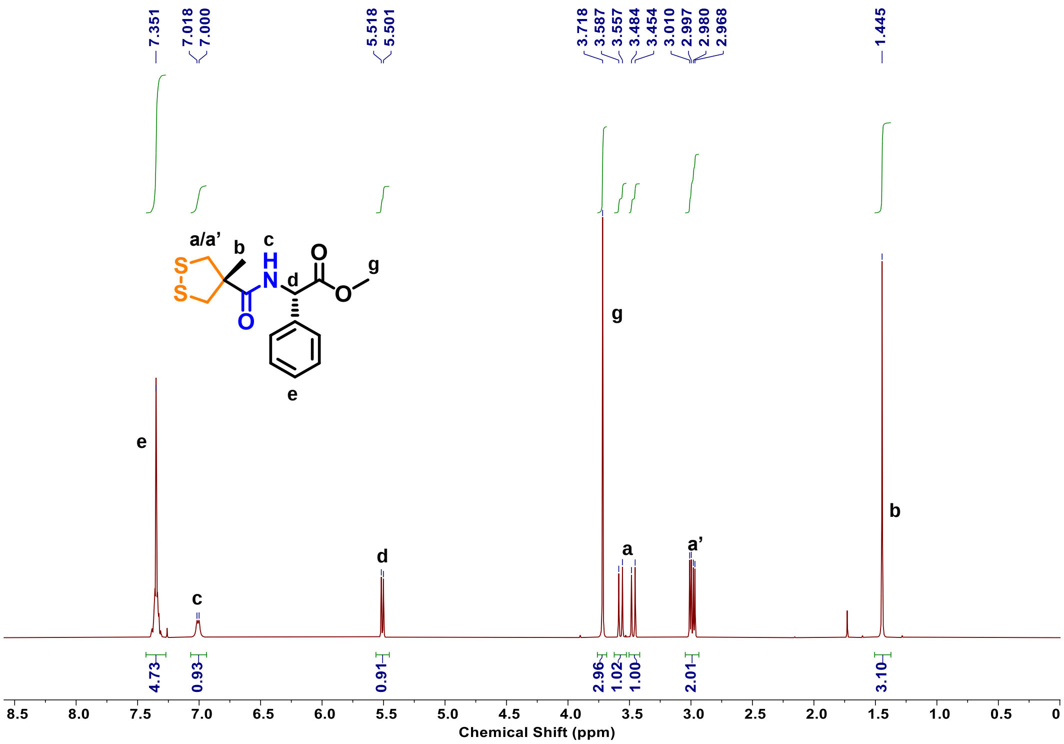

Fig. S29.

^{1}H NMR spectrum of MAA-D-Ben in $CDCl_3$ (400 MHz, 298K).

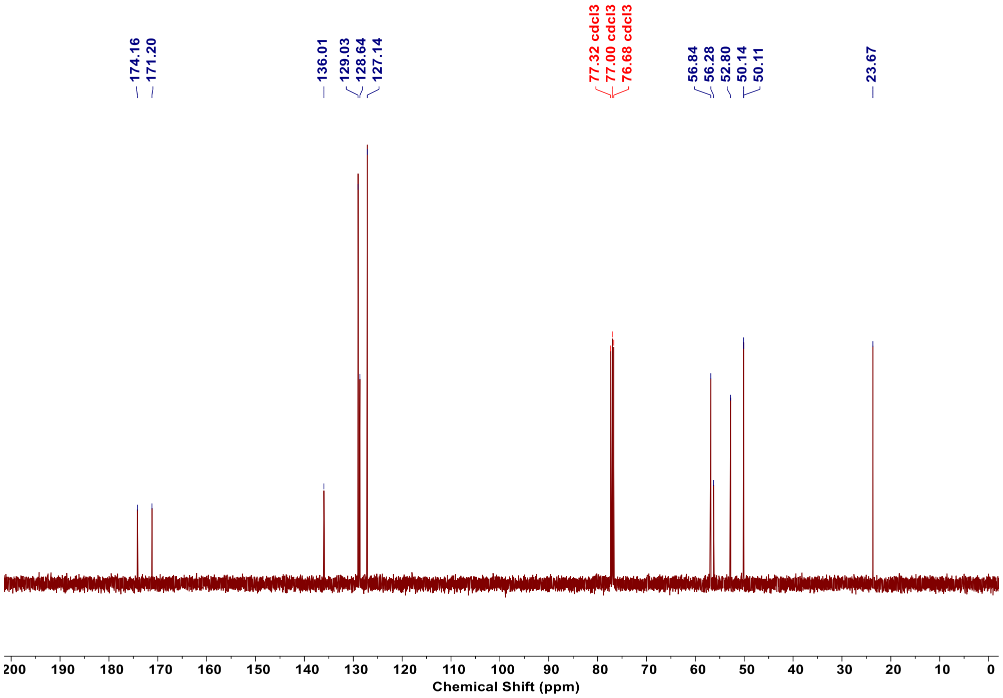

Fig. S30.

^{13}C NMR spectrum of MAA-D-Ben in $CDCl_3$ (101 MHz, 298K).

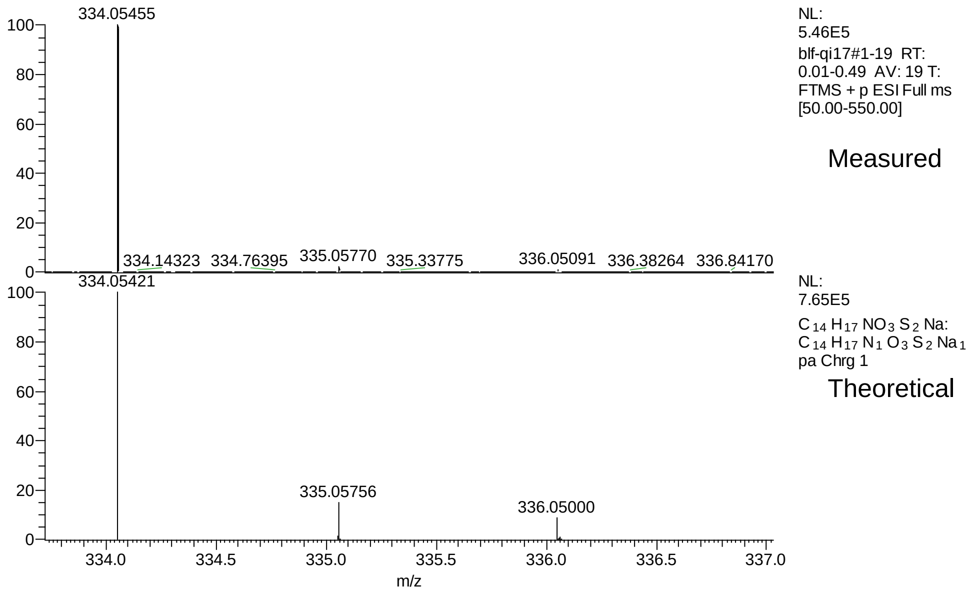

Fig. S31.

HR-MS of MAA-D-Ben (Measured: 334.0546; Calculated: 334.0542).

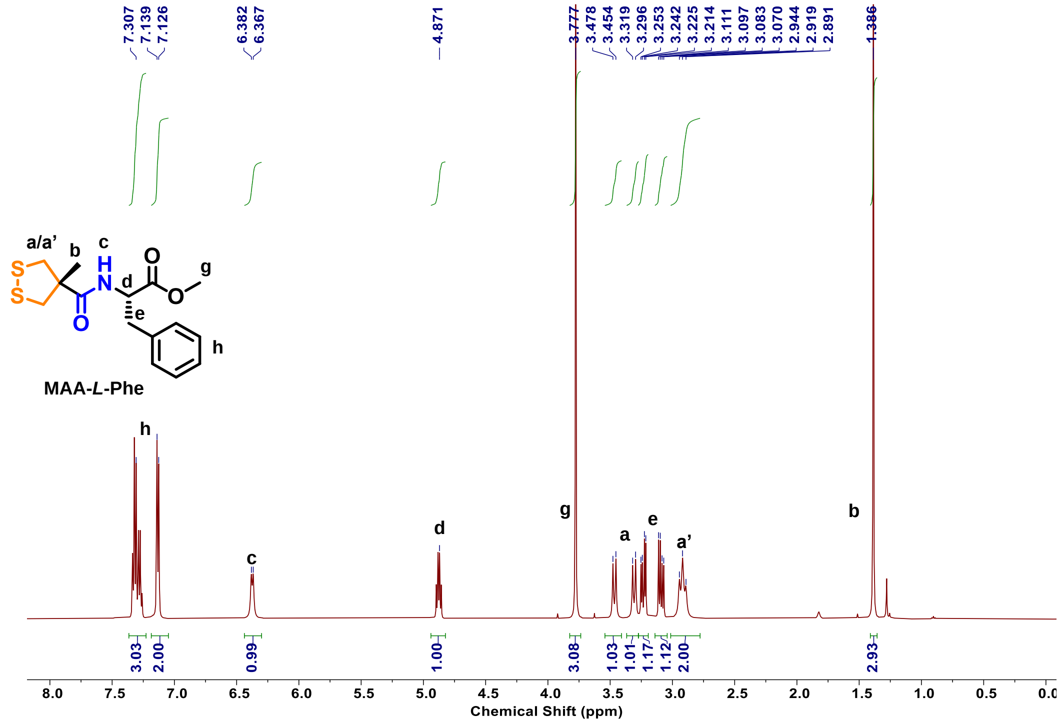

Fig. S32.

^{1}H NMR spectrum of MAA-L-Phe in $CDCl_3$ (400 MHz, 298K).

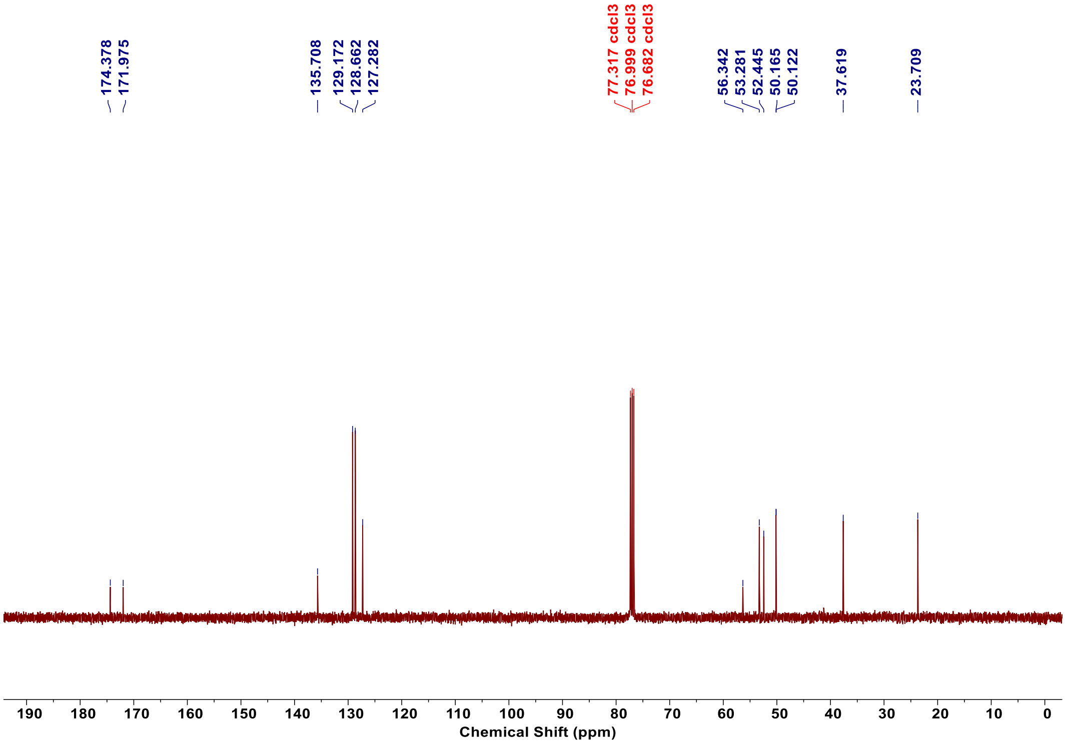

Fig. S33.

^{13}C NMR spectrum of MAA-L-Phe in $CDCl_3$ (101 MHz, 298K).

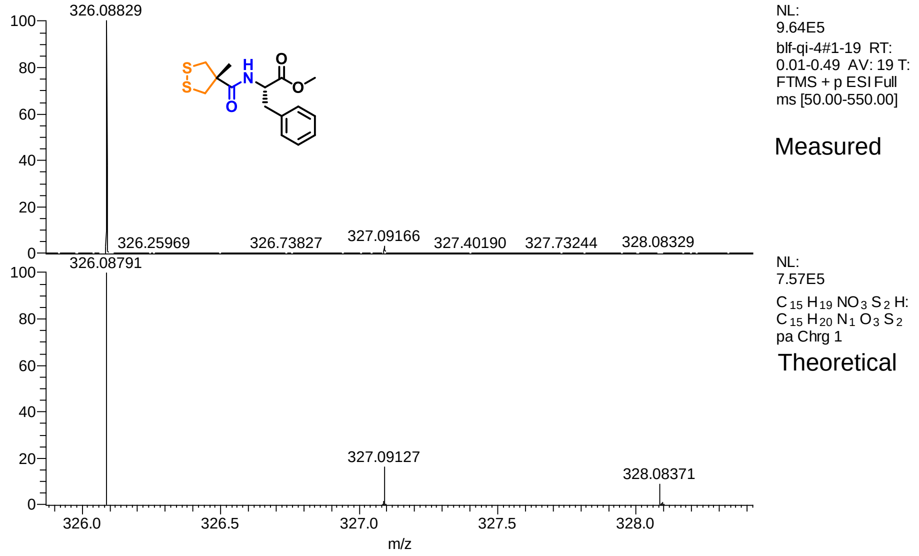

Fig. S34.

HR-MS of MAA-L-Phe (Measured: 326.0083; Calculated: 326.0879).

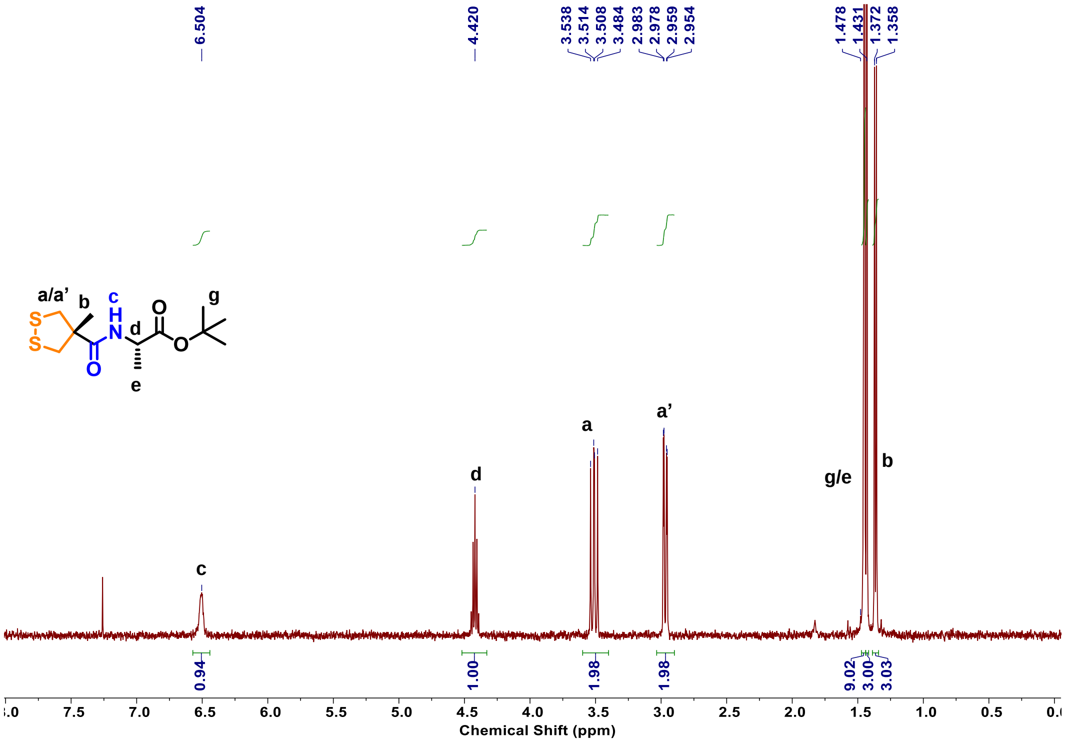

Fig. S35.

^{1}H NMR spectrum of MAA-L-Ala-OBu in $CDCl_3$ (500 MHz, 298K).

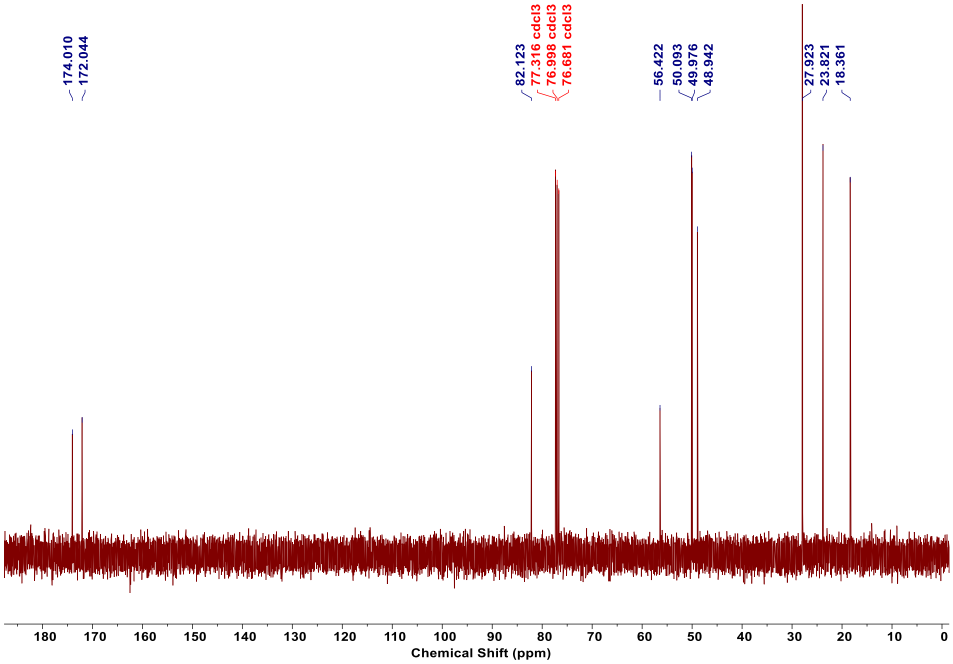

Fig. S36.
^{13}C NMR spectrum of MAA-L-Ala-OBu in $CDCl_3$ (101 MHz, 298K).

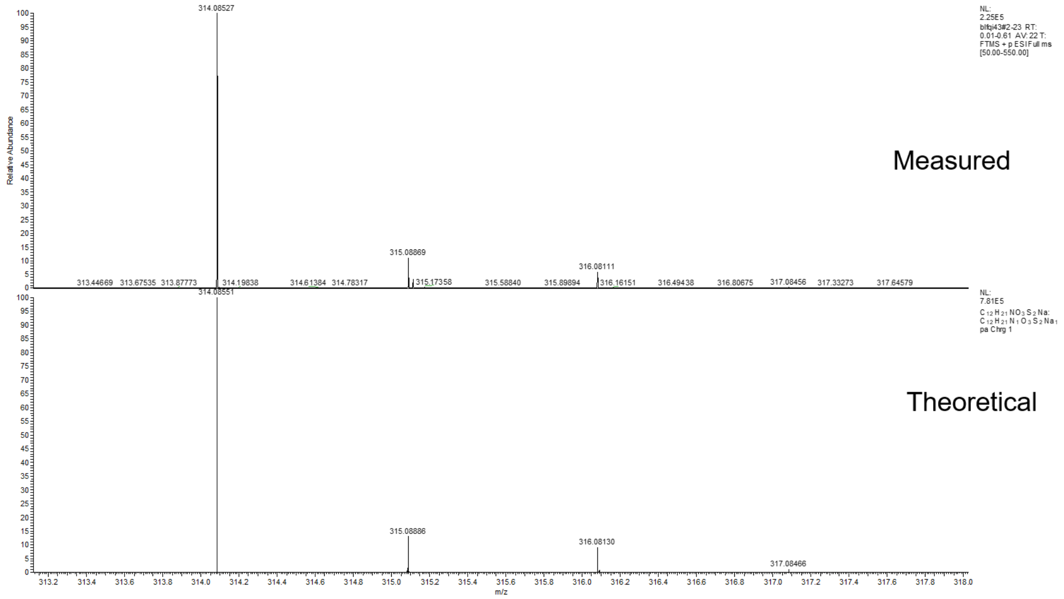

Fig. S37.
HR-MS of MAA-L-Ala-OBu (Measured: 314.0853; Calculated: 314.0855).

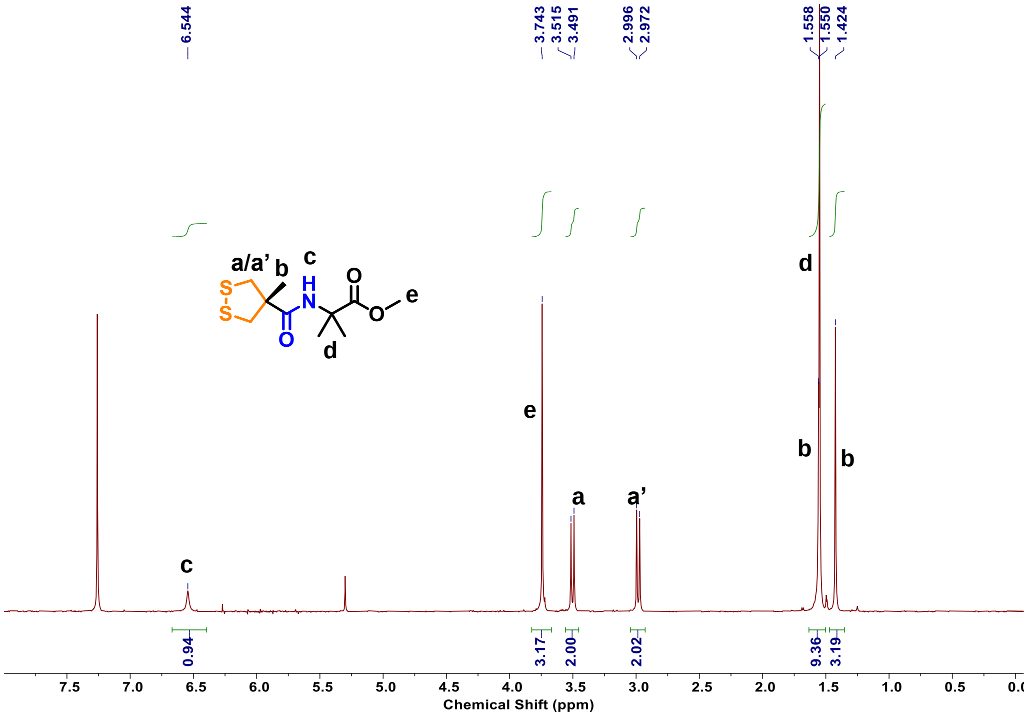

Fig. S38.
^{1}H NMR spectrum of MAA-dmGly in $CDCl_3$ (500 MHz, 298K).

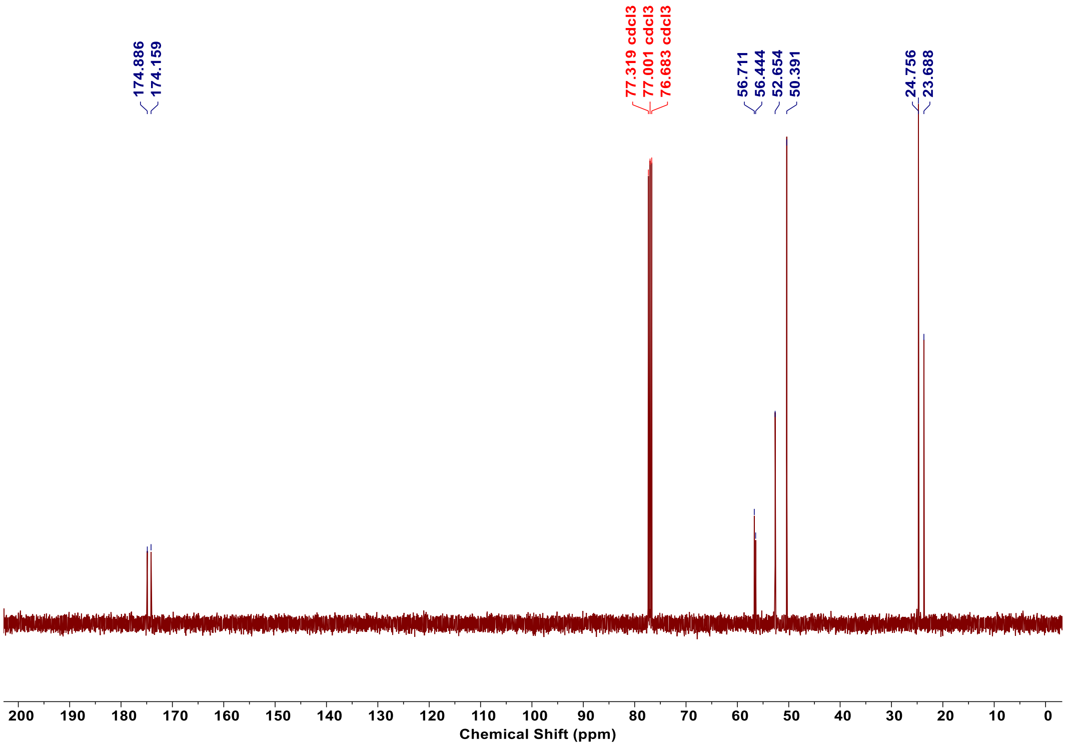

Fig. S39.
^{13}C NMR spectrum of MAA-dmGly in $CDCl_3$ (101 MHz, 298K).

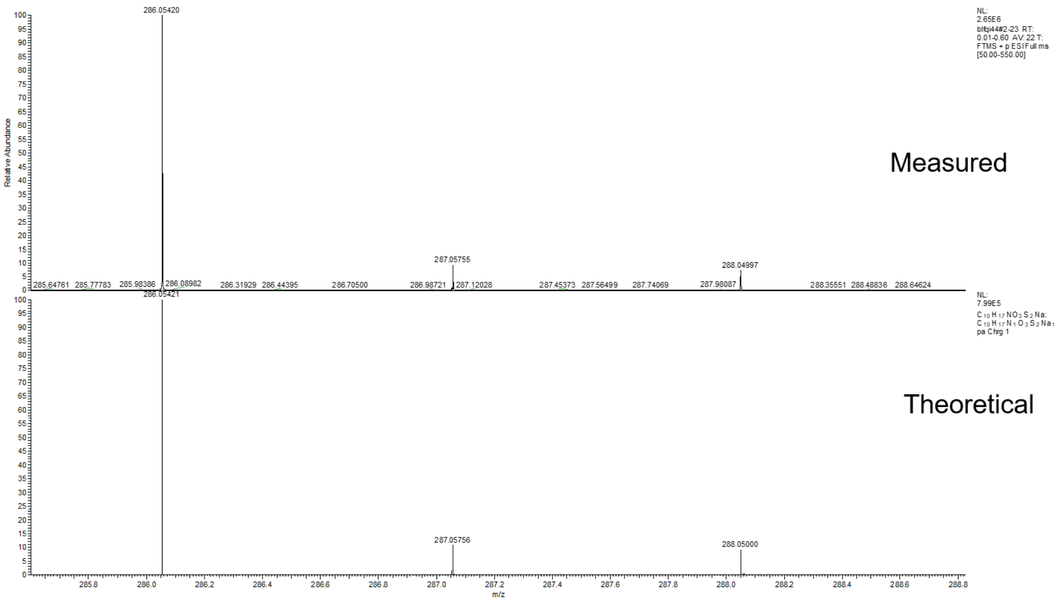

Fig. S40.

HR-MS of MAA-dmGly (Measured: 286.0542; Calculated: 286.0542).

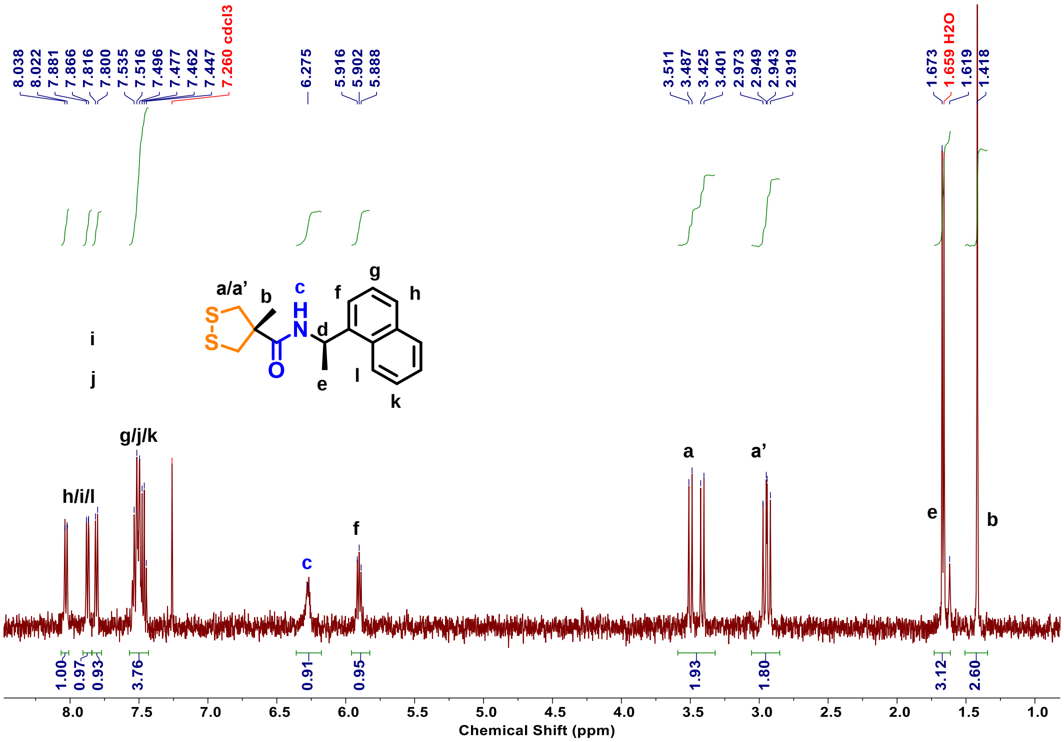

Fig. S41.

^{1}H NMR spectrum of MAA-R-1-NP in $CDCl_3$ (500 MHz, 298K).

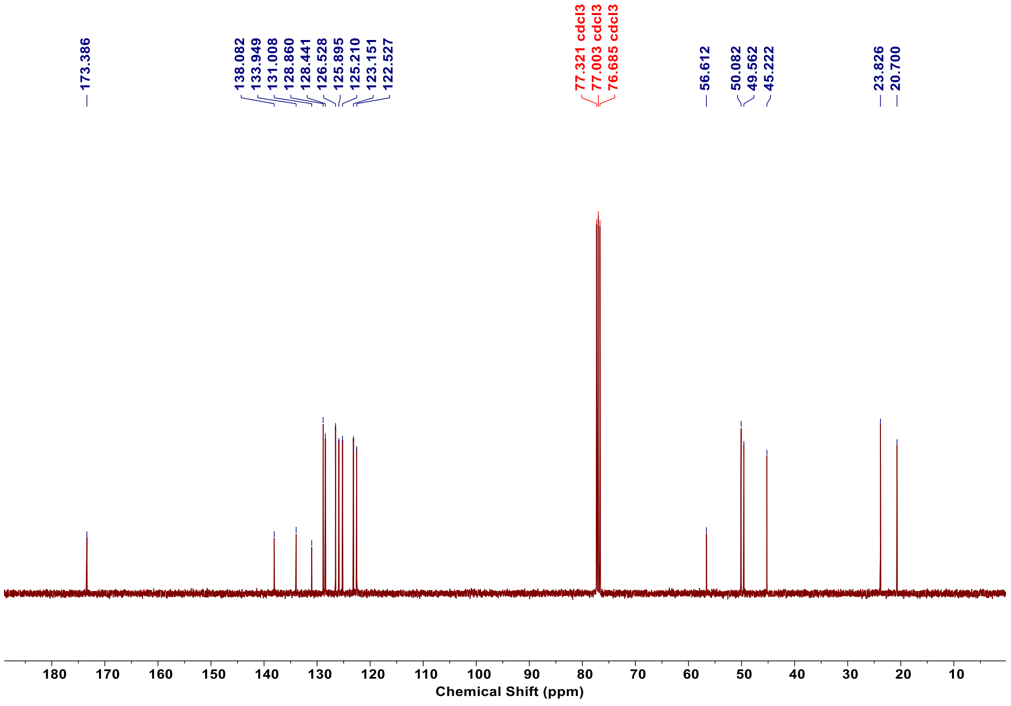

Fig. S42.

^{13}C NMR spectrum of MAA-R-1-NP in $CDCl_3$ (101 MHz, 298K).

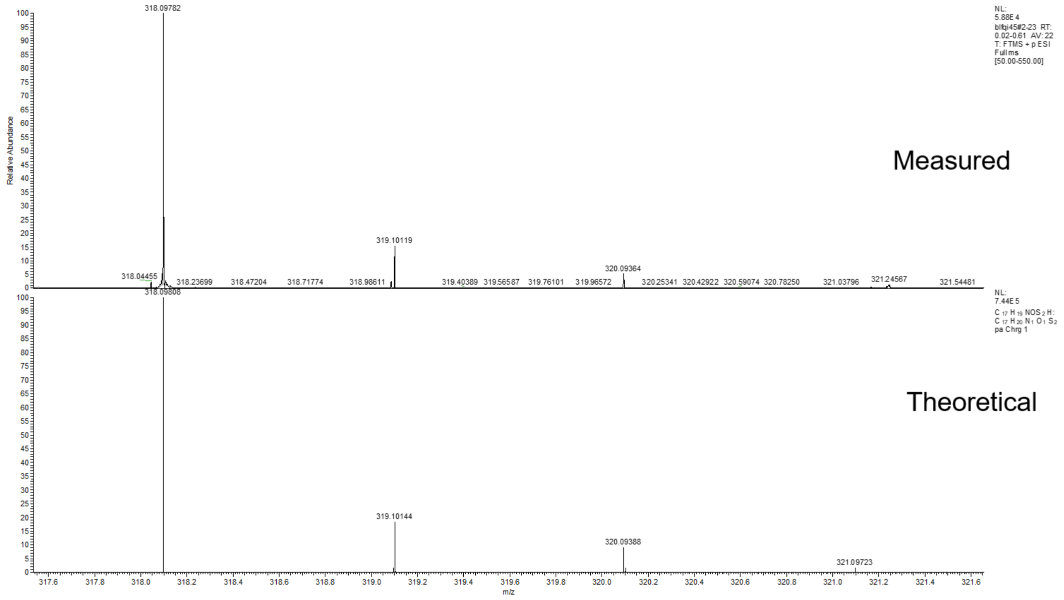

Fig. S43.

HR-MS of MAA-R-1-NP (Measured: 318.0978; Calculated: 318.0981).

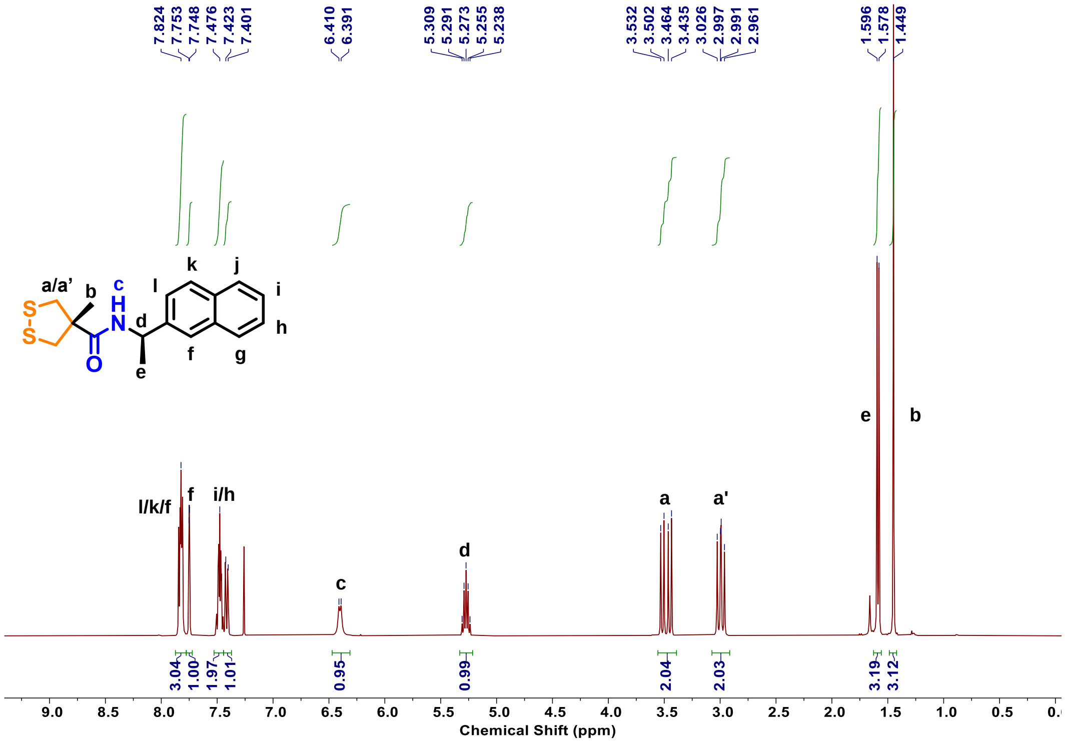

Fig. S44.

^{1}H NMR spectrum of MAA-R-2-NP in $CDCl_3$ (400 MHz, 298K).

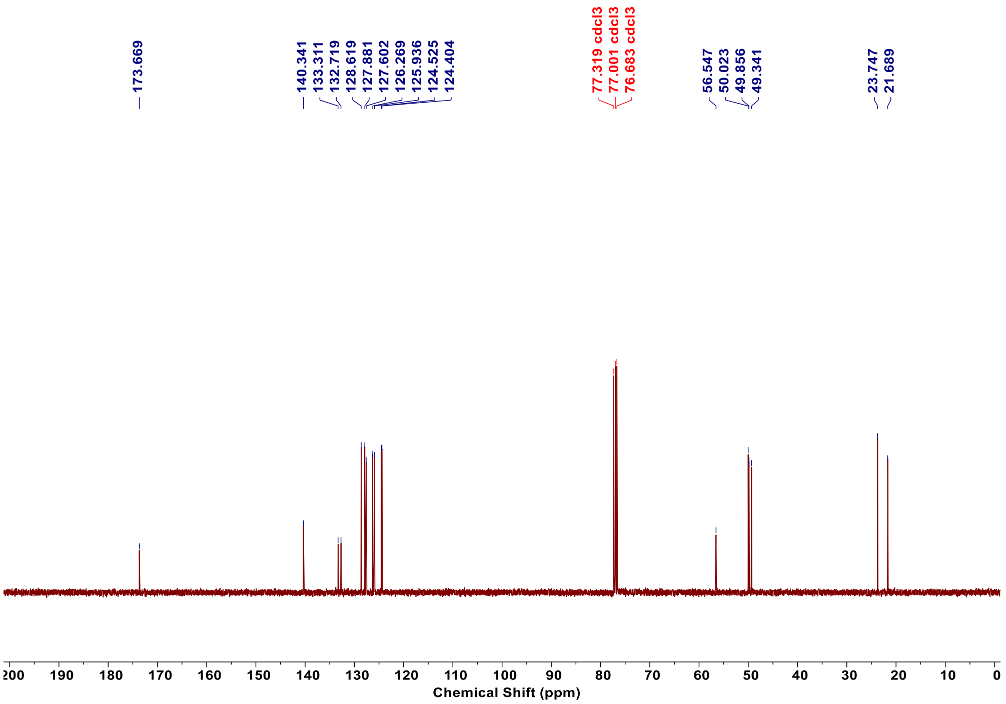

Fig. S45.

^{13}C NMR spectrum of MAA-R-2-NP in $CDCl_3$ (101 MHz, 298K).

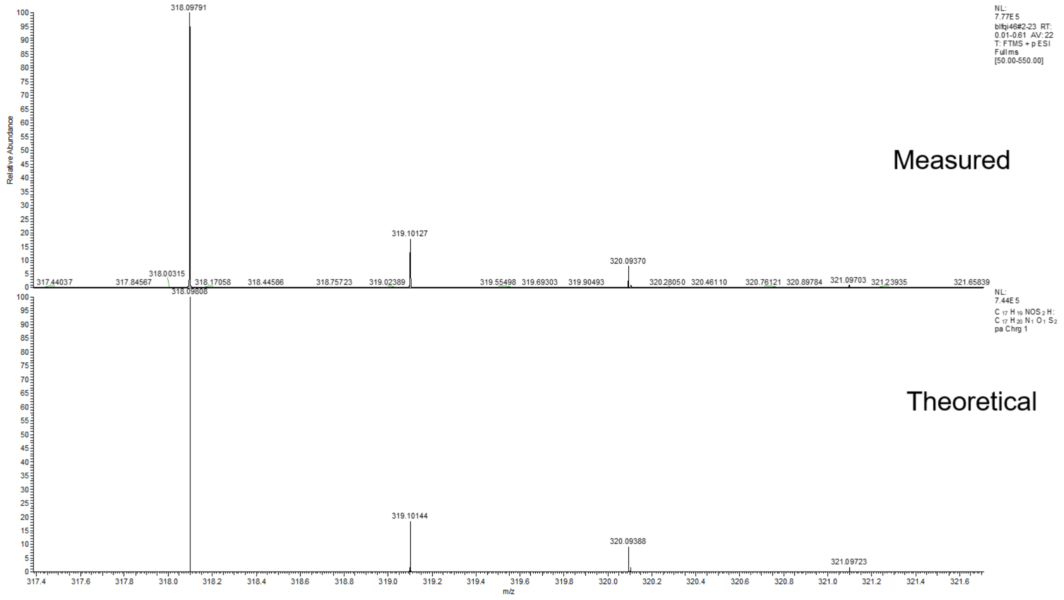

Fig. S46.

HR-MS of MAA-R-2-NP (Measured: 318.0979; Calculated: 318.0981).

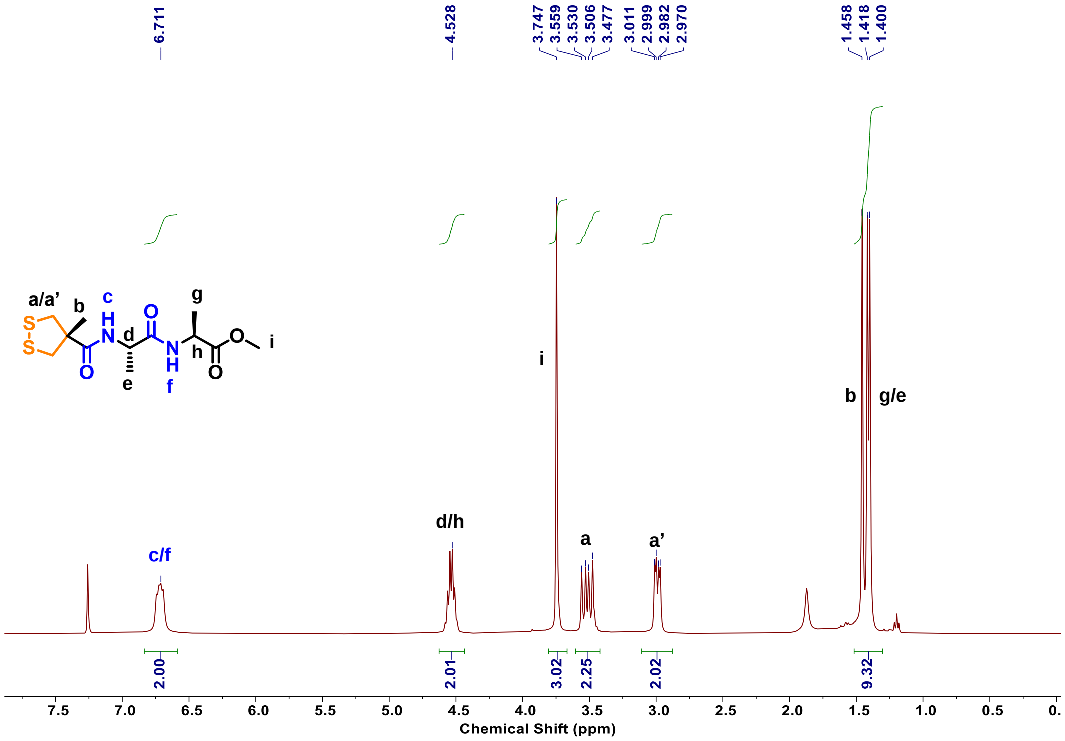

Fig. S47.

^{1}H NMR spectrum of MAA-L-Ala-L-Ala in $CDCl_3$ (400 MHz, 298K).

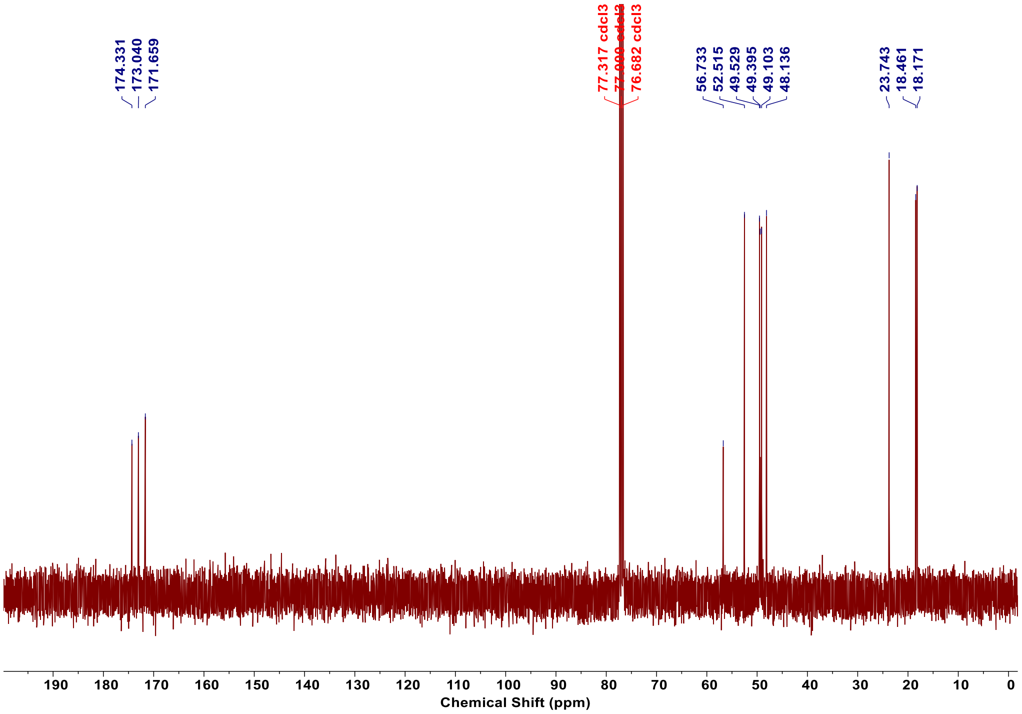

Fig. S48.

^{13}C NMR spectrum of MAA-L-Ala-L-Ala in $CDCl_3$ (101 MHz, 298K).

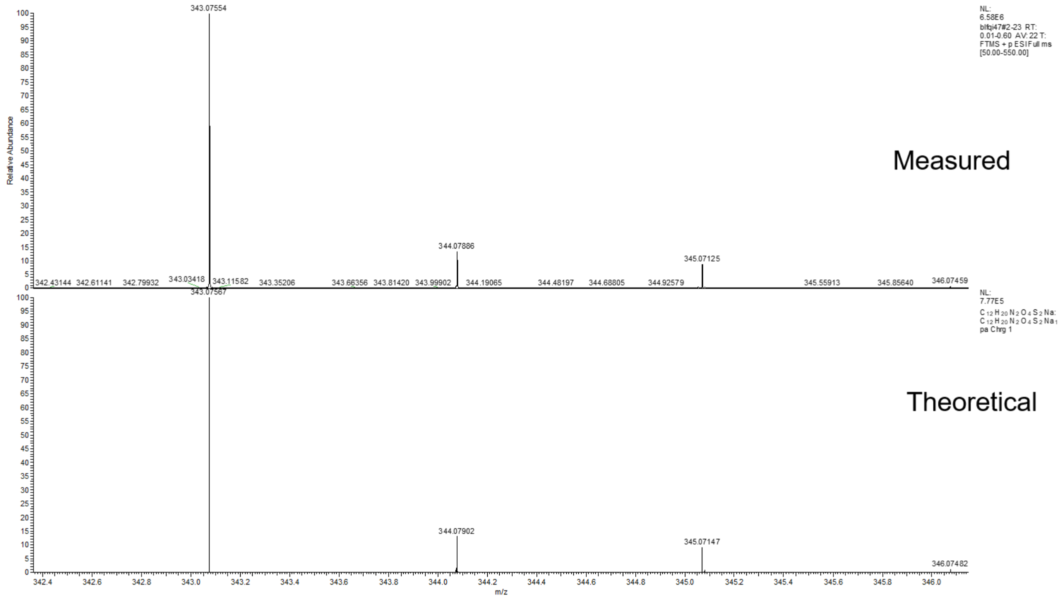

Fig. S49.

HR-MS of MAA-L-Ala-L-Ala (Measured: 343.0755; Calculated: 343.0757).

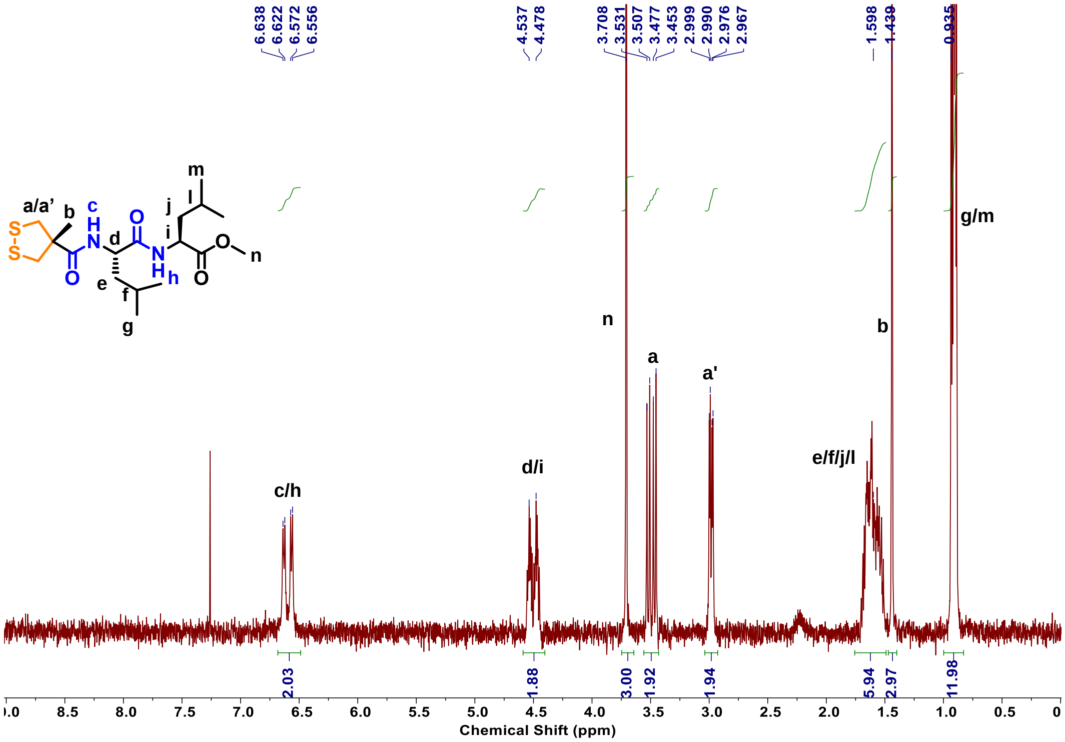

Fig. S50.

^{1}H NMR spectrum of MAA-L-Leu-L-Leu in $CDCl_3$ (500 MHz, 298K).

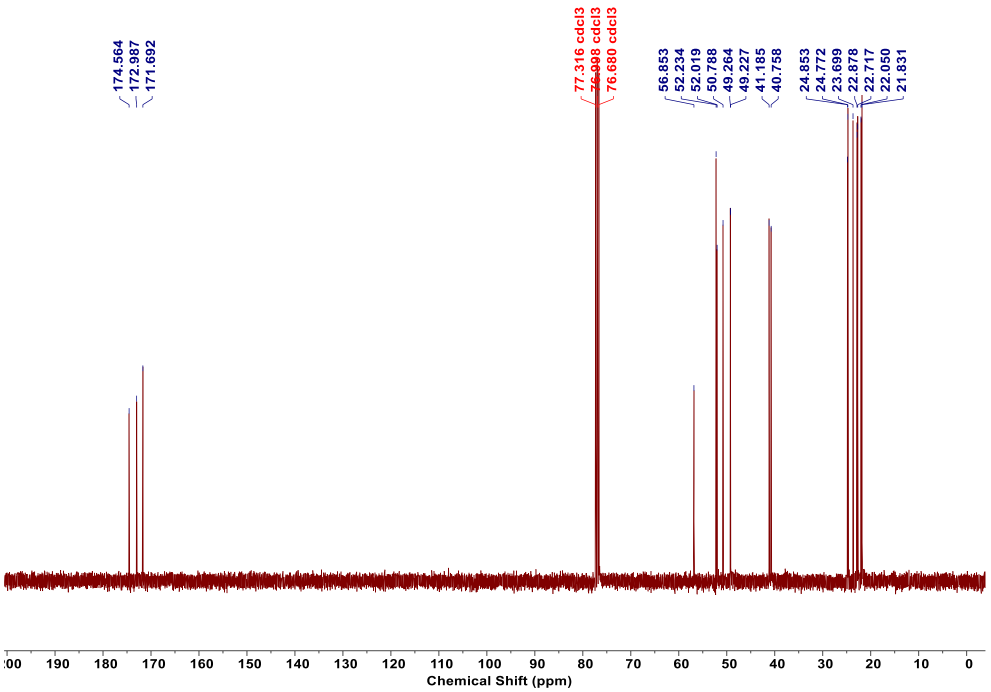

Fig. S51.

^{13}C NMR spectrum of MAA-L-Leu-L-Leu in $CDCl_3$ (101 MHz, 298K).

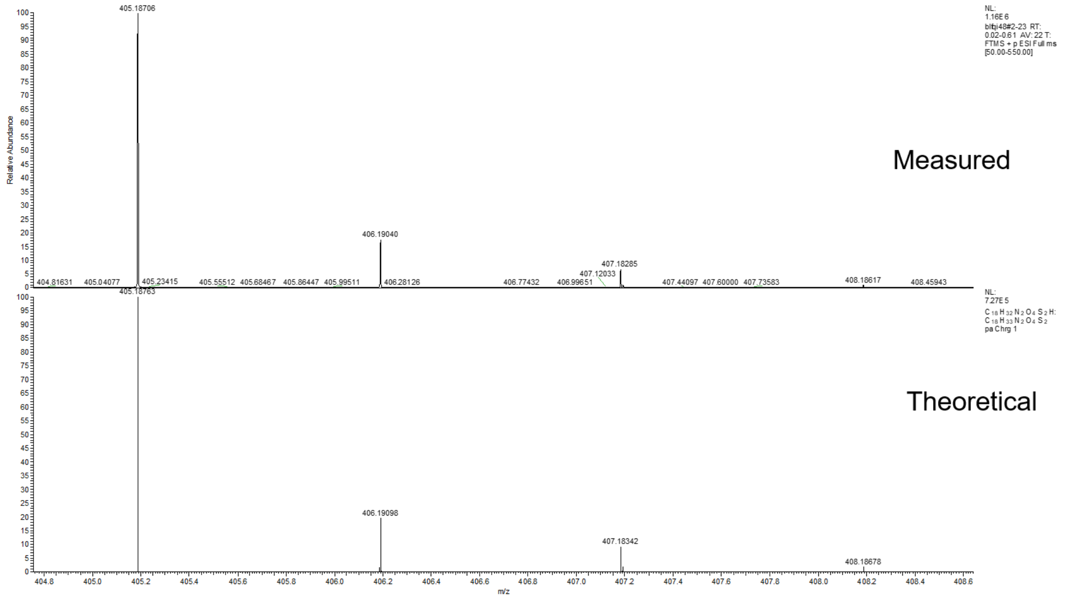

Fig. S52.

HR-MS of MAA-L-Leu-L-Leu (Measured: 405.1871; Calculated: 405.1876).

Supplementary References:

[1] Pracht, P., Bohle, F., Grimme, S. (2020). Automated exploration of the low-energy chemical space with fast quantum chemical methods. Phys. Chem. Chem. Phys. *22*, 7169-7192.

[2] Gaussian 16, Revision B.01, M. J. Frisch, G. W. Trucks, H. B. Schlegel, G. E. Scuseria, M. A. Robb, J. R. Cheeseman, G. Scalmani, V. Barone, G. A. Petersson, H. Nakatsuji, X. Li, M. Caricato, A. V. Marenich, J. Bloino, B. G. Janesko, R. Gomperts, B. Mennucci, H. P. Hratchian, J. V. Ortiz, A. F. Izmaylov, J. L. Sonnenberg, D. Williams-Young, F. Ding, F. Lipparini, F. Egidi, J. Goings, B. Peng, A. Petrone, T. Henderson, D. Ranasinghe, V. G. Zakrzewski, J. Gao, N. Rega, G. Zheng, W. Liang, M. Hada, M. Ehara, K. Toyota, R. Fukuda, J. Hasegawa, M. Ishida, T. Nakajima, Y. Honda, O. Kitao, H. Nakai, T. Vreven, K. Throssell, J. A. Montgomery, Jr., J. E. Peralta, F. Ogliaro, M. J. Bearpark, J. J. Heyd, E. N. Brothers, K. N. Kudin, V. N. Staroverov, T. A. Keith, R. Kobayashi, J. Normand, K. Raghavachari, A. P. Rendell, J. C. Burant, S. S. Iyengar, J. Tomasi, M. Cossi, J. M. Millam, M. Klene, C. Adamo, R. Cammi, J. W. Ochterski, R. L. Martin, K. Morokuma, O. Farkas, J. B. Foresman, and D. J. Fox, Gaussian, Inc., Wallingford CT, 2016.

[3] Bruker, (2016). *APEX3* (v2016.1-0), *SAINT* (Version 8.18C) and *SADABS* (Version 2012/1). Bruker AXS Inc., Madison, Wisconsin, USA.

[4] Sheldrick, G. M. (2015) *Acta Cryst.* **A71**, 3-8.

[5] Sheldrick, G. M. (2008). *Acta Cryst.* A**64**, 112-122.

[6] Zhang, Q., Crespi, S., Toyoda, R., Costil, R., Browne, W. R., Qu, D. H., Tian, H., Feringa, B. L. Stereodivergent chirality transfer by noncovalent control of disulfide bonds. *J. Am. Chem. Soc.* **2022**, *144*, 4376-4382.
